# Practice and proficiency of Isha Yoga for better mental health outcomes: insights from a COVID-19 survey

**DOI:** 10.3389/fpubh.2024.1280859

**Published:** 2024-02-02

**Authors:** Saketh Malipeddi, Seema Mehrotra, John P. John, Bindu M. Kutty

**Affiliations:** ^1^Centre for Consciousness Studies, Department of Neurophysiology, NIMHANS, Bengaluru, Karnataka, India; ^2^Department of Clinical Psychology, NIMHANS, Bengaluru, Karnataka, India; ^3^Multi-modal Brain Image Analysis Laboratory, Department of Psychiatry, NIMHANS, Bengaluru, Karnataka, India

**Keywords:** COVID-19 pandemic, Yoga, meditation, Isha Yoga, mental health, perceived stress, well-being

## Abstract

**Introduction:**

The COVID-19 pandemic has brought about unparalleled suffering on a global scale, affecting both physical and mental well-being. In such challenging times, it becomes crucial to identify interventions that can alleviate negative mental health outcomes, such as stress, while promoting positive mental health outcomes, like well-being. We report the effectiveness of a mind–body practise, Isha Yoga, in promoting well-being.

**Methods:**

We conducted an online survey, during the COVID-19 pandemic, with Yoga practitioners (*n* = 1,352) from the Isha Yoga tradition in Karnataka, India. We evaluated stress and well-being attributes using conventional psychometric questionnaires. Subsequently, we requested the Isha Yoga practitioners to share another survey with their friends and family members, assessing similar outcomes. From the respondents of this shared survey (*n* = 221), we identified individuals who currently did not engage in any form of Yoga or meditation, constituting the non-Yoga control group (*n* = 110). To enhance the reliability and validity of our study and minimize the limitations commonly associated with online surveys, we adhered to the CHERRIES guidelines for reporting survey studies.

**Results:**

Isha Yoga practitioners had significantly lower levels of stress (*p* < 0.001, g_Hedges_ = 0.94) and mental distress (*p* < 0.001, g_Hedges_ = 0.75) while reporting significantly higher levels of well-being (*p* < 0.001, g_Hedges_ = 0.78) and affective balance (*p* < 0.001, g_Hedges_ = 0.80) compared to the control group. Furthermore, expertise-related improvements were observed in these outcomes, and a dose–response relationship was found between regularity of Isha Yoga practice and outcome changes. A minimum 3–4 days of weekly practice showed significant differences with the control group. In addition, we investigated the effect of Isha Yoga on stress and well-being among the healthcare workers (HCWs) in our sample and observed better mental health outcomes.

**Discussion:**

These findings collectively underscore the benefits of Mind and Body practices like Isha Yoga on various aspects of mental health and well-being, emphasizing its potential as an effective and holistic approach for promoting a healthy lifestyle among diverse populations, including healthcare workers, even in difficult circumstances such as the COVID-19 pandemic.

## 1 Introduction

The COVID-19 pandemic has profoundly impacted human well-being worldwide, leading to significant suffering. A large-scale Centers for Disease Control and Prevention (CDC) survey conducted during April–June 2020 in the USA showed a staggering 3- to 4-fold increase in symptoms of anxiety and depression compared to the same period in 2019 (1). This highlights the ongoing mental health pandemic (2). Prior to the pandemic, in 2017, one in seven Indians suffered from mental disorders of varying severity (3). Further, according to the 2016 National Mental Health Survey, the overall prevalence of common mental disorders, including anxiety and depressive disorders, in India was 5.1 percent (4). Though high-quality studies from India during the pandemic are limited (5), a few survey studies reported a significant increase in cases during the pandemic (6, 7). For example, an online survey in India (*n* = 1,871) conducted under the aegis of the Indian Psychiatric Society found that 74.1 percent of all survey respondents had moderate stress, 40.5 percent had anxiety or depression, and 71.7 percent had poor well-being (7). During such challenging times, it becomes crucial to address the need for interventions that promote positive mental health outcomes, including well-being and balance, while mitigating negative outcomes such as stress, anxiety, and depression, ultimately enhancing overall quality of life.

Well-being is defined as “a complex construct that concerns optimal experience and functioning” (8) and includes components of hedonic and eudaimonic well-being. The hedonic aspect, also called subjective well-being, focuses on happiness, higher positive feelings, and lower negative feelings (9, 10). Whereas the eudaimonic aspect, also called psychological well-being, places an emphasis on meaning, purpose and self-actualization (8, 11). The World Health Organization (WHO) closely links health and well-being, defining health as “a state of complete physical, mental, and social well-being, not just the absence of disease or infirmity” (12). The pandemic increased the global prevalence of depression by 27.6 percent (95% UI (uncertainty interval): 25.1–30.3), resulting in an additional 53.2 million cases and anxiety disorders by 25.6 percent (95% UI: 23.2–28.0), resulting in an additional 76.2 million cases worldwide (13). The same study showed that, in South Asia, there has been an increase in the prevalence of major depressive disorder by 36.1% (95% UI: 29.7–42.8) and anxiety disorders by 35.1% (95% UI: 28.2–42.0). Further, a meta-analysis found that the prevalence of depression was 37.12 percent, anxiety was 41.42 percent, and stress was 44.86 percent among health care workers (HCWs) during the pandemic (14). It is quite disturbing to observe such a high prevalence of negative symptoms that impair human quality of life, well-being, and social functioning, ultimately affecting humanity itself.

Mind and Body practices (MBPs) such as Yoga and meditation have emerged as popular, credible, and effective interventions in promoting positive outcomes and reducing negative outcomes (15, 16). Mindfulness meditation has been extensively studied and shown to reduce stress and improve well-being (17). Other practices such as Heartfulness, Raja Yoga, and Transcendental Meditation have also reported similar beneficial effects (18–20). Notably randomized controlled trials (RCTs) further reinforce the findings of earlier research (21, 22). A growing body of evidence suggests a link between Yoga practises and lower levels of stress, anxiety, and depression, as well as greater well-being (23–28). However, it is essential to recognize that the primary purpose of Yoga lies not in these incidental benefits, but rather to provide a path to overcoming suffering and realizing one’s true nature. In India, diverse traditions of Yoga exist, among which is Isha Yoga.

Isha Yoga, an international school of Yoga, offers a holistic system that integrates all four paths of Yoga – Karma, Bhakti, Gnana, and Kriya – and provides methods to promote well-being. Each of these practices is designed to enhance physical, mental, and emotional well-being (26, 29–32). Studies with Isha Yoga practices reported reduced stress levels, increased mindfulness, and enhanced well-being (23, 25, 33, 34), improved cardiac sympatho-vagal balance (29), heightened visual attention (35), elevated levels of BDNF and CAR (30), increased anandamide levels (36), enhanced immune system functioning indicated by higher anti-viral interferon gene expression (37), elevated EEG gamma power during meditation (38), and so on. Several survey studies were conducted among Isha Yoga practitioners during the COVID-19 pandemic, and they reported lower negative mental health outcomes and higher positive mental health outcomes, showing its potential as a useful mind–body intervention (23–25, 34). However, some of these studies had low sample sizes. Further, no studies were carried out in the Indian population, requiring the need for the present study.

Moreover, we have been conducting research to assess the neuroscientific aspects of Isha Yoga practices for the last five years. We were able to demonstrate various trait and state effects of Isha Yoga practice among the advanced practitioners, including unique brain oscillatory dynamics indicative of “relaxed alertness,” greater attention as indexed by a greater P300 amplitude, and higher well-being and lower perceived stress scores (manuscript in preparation). To demonstrate the generalizability of these findings to a larger population of Isha Yoga meditators, we conducted the current online cross-sectional survey, during the COVID-19 pandemic, using a large sample of more than 1,300 Isha Yoga practitioners. Our aim was to address gaps in research and provide additional insights into the effects of Isha Yoga practises among Indian participants. Based on the existing literature on Isha Yoga, other meditative traditions, and what we observed in our EEG study, we began the online survey with the hypothesis that practising Isha Yoga would be associated with lower negative outcomes (perceived stress and mental distress) and higher positive outcomes (well-being and balance). Furthermore, we hypothesized that the practitioner’s expertise and proficiency would be critical in influencing outcome measures.

Finally, it is essential to recognize that internet/web-based surveys often come with methodological concerns like non-representativeness of the sample, self-selection bias, non-response bias, and so on, that can potentially affect their validity (39, 40). To ensure the rigor and reliability of our study and to overcome some of the above-mentioned limitations, we adhered to appropriate guidelines (41).

## 2 Methods

### 2.1 Participant recruitment and sampling methodology

This study was conducted as a cross-sectional online survey using Google Forms in the southern Indian state of Karnataka from August to October 2021 (survey provided in the Supplementary file). By October 2021, the total active COVID-19 cases in the state were 9,621, and the total cases were 29,82,089 (42). Also, a large proportion of the Karnataka adult population (75% by December 2021) had been vaccinated with both doses (43). The government had also eased the lockdown measures as the number of cases dropped. During this period, Isha Foundation helped us in distributing the survey among its meditator population in Karnataka. Individuals who had participated in at least one Isha Yoga program, such as Hatha Yoga or Inner Engineering, were referred to as Isha Yoga practitioners. The Isha Foundation maintains a database of all such individuals, and in Karnataka, this number was 40,682 as of October 2021. This population was considered the target population, and an address-based sampling strategy was employed. All the individuals in the target population were contacted via email in two separate campaigns conducted in August and October. Advertisement to contact the Isha meditators is provided in the Supplementary file. Information regarding the number of emails sent, the number of unique opens, and the number of unique clicks was obtained from Google Analytics data.

Survey statistics for Isha meditators are given below:

**Table:** 

Month	Mails sent to	Unique opens	Unique clicks
August	39,833	5,977 (15%)	690
October	40,682	9,611 (24%)	1,200

Total unique opens: 15,588.

Total unique clicks: 1,890.

Total responses: 1,352.

Participation rate: 71.53% [(Total responses/Total unique clicks) *100].

The CHERRIES guidelines (41) (Table 1) were followed in conducting the survey, ensuring adherence to appropriate standards for reporting the results of internet-based surveys.

**Table 1:** The checklist for reporting results of internet E-surveys (CHERRIES).

Item category	Checklist item	Explanation
Design	Describe survey design	Target population: Isha Yoga practitioners in Karnataka Controls: Nominated by Isha Yoga practitioners and people known to the study authors Sampling type: Isha Yoga – Address based sampling Controls – Convenience and snow-ball sampling
IRB (Institutional Review Board) approval and informed consent process	IRB	This study was approved by the NIMHANS human ethics committee (NIMH/DO/ETHICS SUB-COMMITTEE MEETING/2018)
	Informed Consent	Consent was taken from all the study participants
	Data protection	All the personal details taken from the survey respondents remains with the study authors. No one has access to this. Data is password-protected.
Development and pre-testing	Development and testing	Some of the questions in the survey were developed following feedback from experts in the field of Yoga and well-being. Whereas, other questions are from already validated psychological questionnaires like PSS, WHO-WBI, etc.
Recruitment process and description of the sample having access to the questionnaire	Open survey versus closed survey	Open survey
	Contact mode	By mail
	Advertising the survey	Surveys were sent to the Isha Yoga practitioners in Karnataka. Isha Foundation helped the study authors to reach out to the participants. Survey announcement is provided in the Supplementary data. Mailing list: Isha Yoga practitioners: Yes, database of Isha practitioners in Karnataka Controls: No, nominated by Isha practitioners and others known to authors
Survey administration	Web/E-mail	By Email. Responses were captured automatically by google forms.
	Context	Surveys were sent to a clearly defined target population of Isha Yoga practitioners in Karnataka. Because of this, sample is highly likely to be representative of the Isha Yoga population in Karnataka.
	Mandatory/voluntary	It was a voluntary survey
	Incentives	No incentives were provided for participating in the survey
	Time/Date	Timeframe for data collection: August to October, 2021
	Randomization of items or questionnaires	Items were not randomized or alternated
	Adaptive questioning	No, this was not followed
	Number of Items	Number of questionnaire items per page were in the range 6–10
	Number of screens (pages)	Number of pages were 7
	Completeness check	All the questions in the survey were mandatory. Wherever appropriate, items had non-response options such as “not applicable” or “prefer not to say.” This way, selection of one response was enforced.
	Review step	Respondents could edit their responses while filling the survey. This would not be possible once they complete the survey.
Response rates	Unique site visitor	Google analytics data helped us capture the number of people with unique clicks and unique opens.
	View rate (Ratio of unique survey visitors/unique site visitors)	Isha Yoga practitioners: 12.12% [(1,890/15,588) * 100] Controls: Cannot be calculated because of sampling type
	Participation rate (Ratio of unique visitors who agreed to participate/unique first survey page visitors)	Isha Yoga practitioners: 71.53% [(1,352/1,890) * 100] Controls: Cannot be calculated because of sampling type
	Completion rate (Ratio of users who finished the survey/users who agreed to participate)	Isha Yoga practitioners: 98.47% [(1,352/1,373) *100] Controls: 96.92% [(221/228) *100]
Preventing multiple entries from the same individual	Cookies used	Cookies were not used. Duplicate entries were prevented by making it mandatory for the survey respondents to enter their email address.
	IP check	No
	Log file analysis	No
Analysis	Handling of incomplete questionnaires	Completion rates were high. All questions were mandatory.
	Questionnaires submitted with an atypical timestamp	This was not done
	Statistical correction	Tests were corrected for multiple comparisons using Holm’s method

Additionally, Isha Yoga meditators were requested to nominate at least one friend or family member and share another survey with them (Supplementary sheet). This group was intended to serve as a control group for comparison. A combination of convenience sampling and snowball sampling strategies was employed to gather responses from these groups. In total, 221 responses were received from these sources. Questions 16–19 in the survey for Isha nominees were included to ensure that people who had never practised Yoga or meditation (*n* = 110) were included as controls for the Isha Yoga practitioners. The practice characteristics of these participants are provided in Supplementary Table S2.

### 2.2 Inclusion and exclusion criteria

#### 2.2.1 For Isha Yoga practitioners

The inclusion criteria for participation in the study were:

- Practicing only Isha Yoga
- Age ≥ 18
- Ability to read and understand English
- Resident of Karnataka

#### 2.2.2 For controls

The inclusion criteria for being part of the sample were:

- Currently practice no form of Yoga or meditation
- Age ≥ 18
- Ability to read and understand English
- Resident of Karnataka

### 2.3 Isha Yoga

Isha Yoga trains people to achieve inner well-being. A unique aspect of Isha Yoga is that it does not have any philosophy or belief system. Isha Yoga encompasses various practices like Shambhavi Mahamudra Kriya, Shoonya, and Samyama (26, 31, 44). Shambhavi Mahamudra Kriya is offered as part of the Inner Engineering program, which is designed to help individuals explore and enhance their inner well-being. Inner Engineering has two components: Inner Engineering Online (IEO) and Inner Engineering Completion (IEC) (32, 33). IEO, a 4-week self-paced program, consists of seven 90-min sessions that provide participants with practical techniques and insights to manage their physical, mental, and emotional well-being. It promotes intellectual inquiry and includes Upa Yoga (preparatory Yoga involving body movement and breath practices). IEO is a pre-requisite for IEC, where Shambhavi Mahamudra Kriya is taught. It is a 21-min multi-component practice involving pranayama (breath modulation), AUM chanting, bandhas (engagement of muscular locks), and breath watching meditation (23, 44). Inner Engineering is a foundational program in Isha. Only after being initiated into Shambhavi Mahamudra Kriya is one eligible for advanced programs such as Shoonya and Samyama. Shoonya Meditation in Isha Yoga is an advanced meditation practice. The term “Shoonya” translates to “no-thingness.” It is a “conscious non-doing” practice that is to be practised for 15-min, twice daily. This meditation technique aims to help individuals experience a state of inner stillness (38). Samyama is an intensive residential program that spans eight days and is conducted in an environment of complete silence. It is an advanced practice that combines two fundamental spiritual processes in the Yogic tradition: Pragna, the path of awareness, and Samadhi, the path of dissolution (44).

### 2.4 Outcome measures

Each participant was asked to respond to 4 validated psychological questionnaires assessing-

- Negative outcomes: stress (Perceived Stress Scale, PSS) and mental distress (anxiety and depression) (PHQ-4)
- Positive outcomes: subjective well-being (WHO-WBI) and balance (SPANE-B)

The details of the questionnaires are given below.

#### 2.4.1 Perceived Stress Scale

The Perceived Stress Scale (PSS) is a well-established tool for assessing individual’s perceived stress over the past month and consists of 10 items. It is known for its brevity and user-friendly nature (45). Participants respond to each item using a 5-point Likert-type scale, ranging from 0 = never to 4 = very often. The final score is obtained by summing all the item responses and can range from 0 to 40, with higher scores indicating higher perceived stress levels. Interpretation: a score of 0–13 indicates low stress, 14–26 indicates moderate stress, and 27–40 indicates high stress. The internal reliability of the data was assessed using Cronbach’s alpha, and the calculated value was 0.86.

#### 2.4.2 The four-item Patient Health Questionnaire for anxiety and depression (PHQ-4)

The PHQ-4 (Patient Health Questionnaire-4) is designed to assess anxiety and depression in the general population (46). It consists of only four questions, with two items measuring anxiety and the other two measuring depression. Each item is rated on a 5-point Likert-type scale, ranging from 0 = Not at all to 4 = Nearly every day. The total score is obtained by summing the scores for each of the four items. A PHQ-4 score falling within the range of 0–2 indicates normal mental distress, 3–5 suggests mild mental distress, 6–8 indicates moderate mental distress, and 9–12 suggests severe mental distress. Anxiety is calculated by summing the scores for items 1 and 2, with a total score of 3 or higher indicating the presence of anxiety. Similarly, depression is calculated by summing the scores for items 3 and 4, and a total score of 3 or higher suggests the presence of depression. The internal reliability of the data was assessed using Cronbach’s alpha, and the calculated value was 0.8.

#### 2.4.3 Scale of Positive and Negative Experience (SPANE)

The SPANE (Scale of Positive and Negative Experience) is a 12-item scale that measures positive feelings (SPANE-P), negative feelings (SPANE-N), and affect balance (SPANE-B) (10). Participants rate the frequency of experiencing each item on a scale ranging from 1 “Very rarely or never” to 5 “Very often or always.” The SPANE-P score is derived by summing the scores for the six positive items, resulting in a range of 6 (lowest) to 30 (highest) for positive feelings. Similarly, the SPANE-N score is obtained by summing the scores for the six negative items, also ranging from 6 to 30 for negative feelings. The SPANE-B score is calculated by subtracting the SPANE-N score from the SPANE-P score, yielding a difference score ranging from −24 (the lowest affect balance) to 24 (the highest affect balance). A score close to 24 indicates that an individual “very rarely or never” experiences negative feelings and “very often or always” experiences positive feelings, suggesting a high level of well-being and affective balance. The internal reliability of the data was evaluated using Cronbach’s alpha, and the resulting coefficient was 0.92.

#### 2.4.4 WHO Well-Being Index (WHO-WBI)

The World Health Organization Well-Being Index (WHO-5), developed in 1998, is a concise questionnaire consisting of five items to assess the subjective well-being of individuals (47). Respondents rate each item on a scale ranging from 5 “all of the time” to 0 “at no time.” To calculate the raw score, the individual’s responses for all five items are summed. The raw score can range from 0 to 25. Percentage scores are obtained by multiplying the raw scores by 4, resulting in scores that vary from 0 (indicating the lowest level of well-being) to 100 (representing the highest level of well-being). The internal reliability of the data was evaluated using Cronbach’s alpha, and the resulting coefficient was 0.9.

#### 2.4.5 Others

In addition, the survey included various questions, covering aspects such as participants sleep patterns, physical activity levels, dietary habits, medical history, and the impact of COVID-19. For the Isha Yoga practitioners, specific questions were included to assess their daily practices (questions 13–18 in the Isha meditators survey provided in the Supplementary file). This included questions about the types of practices they engage in regularly, the frequency and duration of their daily practice, the total number of hours they have practiced in their lifetime, and their self-rated proficiency in their practice. After consultation with experts in the Isha Yoga tradition, these questions were utilized to categorize the Isha meditators into different levels of expertise, such as novice practitioners (NOV), intermediate practitioners (INT), and advanced practitioners (ADV), as outlined in Supplementary Table S3. The survey for the Isha Yoga nominees included questions pertaining to their engagement with Yoga and meditation practices. This was done to ensure that individuals who currently practice other forms of Yoga were excluded from the analysis, thus maintaining a clear distinction between the Isha Yoga practitioners and the no-Yoga (control) group. Isha Yoga practitioners were also grouped based on practice regularity: Regular practitioners (≥5 times/week, *n* = 1,097); moderately regular practitioners (3–4 times/week, *n* = 139); and Irregular practitioners (<3 times/week, *n* = 108). These cut-offs were determined based on a previous study that used similar grouping (31). These categorizations allowed for a more detailed examination of the impact of different levels of practice regularity within the Isha Yoga practitioner population.

### 2.5 Statistical analysis

The statistical analyses were performed using RStudio Version 1.4.1106. RStudio provides a user-friendly integrated development environment for R, offering efficient data manipulation, visualization, and statistical analysis capabilities. Several R packages were utilized, including ggplot2 (48), ggsci (49), ggstatsplot (50), dplyr (51), effectsize (52), and performance (53), for conducting the necessary analysis. The selection of these R packages was driven by their established reputation for robust analysis and compatibility with the nature of our dataset and research objectives. Descriptive statistics such as mean, median, and standard deviation (SD) were calculated as appropriate for the data. Parametric tests were employed throughout as the sample sizes were large (54, 55). Between-group comparisons were conducted using Welch’s one-way ANOVA. Post-hoc analyses were performed to further examine significant differences between groups using the Games-Howell test. These post-hoc tests aimed to minimize type 1 errors or false positives. Multiple comparisons were adjusted using Holm’s method.

Effect size estimation was carried out using measures such as hedges g, omega squared tests and Cramer’s V. Effect sizes were reported with 95% confidence intervals. The practical significance of effect sizes is crucial for understanding the real-world implications of observed differences or relationships in a study. While statistical significance indicates whether an observed result is likely due to chance, effect sizes provide information about the magnitude or strength of that result. A large effect size suggests that the observed phenomenon has a substantial impact, while a small effect size indicates a more modest influence. For example, in medical research, a treatment might be statistically significant but have a small effect size, raising questions about its meaningful impact on patient outcomes. A significance level of *p* < 0.05, a standard in statistical hypothesis testing, was considered significant, and exact *p* values were reported accordingly. All statistical reporting adhered to the guidelines provided by the American Psychological Association (56), which were followed in the ggstatsplot package (50). An example is given below.


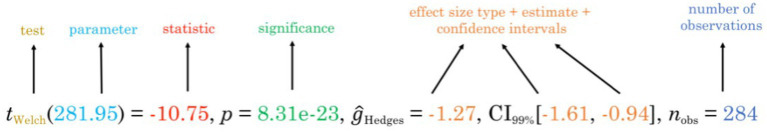
Categorical data (sleep quality and quantity, diet, and physical activity levels) were presented in the form of percentages and analyzed using the Chi-Squared test. A significance level of *p* < 0.05 was considered statistically significant. A 95% confidence interval was calculated and reported alongside the effect size to provide a range of plausible values for the population parameter.

## 3 Results

### 3.1 Participants

Supplementary Table S4 provides the comparison of Isha meditators and Isha Yoga nominees (all the survey respondents). Table 2 provides the subject characteristics for the final sample.

**Table 2:** Subject characteristics: final sample.

Characteristics	Meditator group	Controls (no-Yoga group)
Total sample (*N*)	1,097	110
Age-range (in years):	*n* (%)	*n* (%)
18–29	249 (22.69%)	53 (48.18%)
30–44	559 (50.95%)	42 (38.18%)
45–64	251 (22.88%)	14 (12.72%)
65 years or above	38 (3.46%)	1 (0.9%)
Gender		
Male	626 (57.06%)	39 (35.45%)
Female	470 (42.84%)	71 (64.54%)
Prefer not to say	1 (0.09%)	0
Education		
High school or some college	37 (3.37%)	16 (14.54%)
Bachelor’s degree or equivalent	557 (50.77%)	39 (35.45%)
Postgraduate degree	486 (44.30%)	52 (47.27%)
Prefer not to say	17 (1.54%)	3 (2.72%)
Alcohol consumption		
Yes	47 (4.28%)	7 (6.36%)
Social drinker	160 (14.58%)	20 (18.18%)
No	890 (81.13%)	83 (75.45%)
Smoking		
Yes	54 (4.92%)	7 (6.36%)
No	1,043 (95.07%)	103 (93.63%)
Substance use in the last 6 months		
Yes	28 (2.55%)	4 (3.63%)
No	1,069 (97.44%)	106 (96.36%)

### 3.2 Mental health outcomes: overall differences

#### 3.2.1 Perceived stress

**Figure 1:**
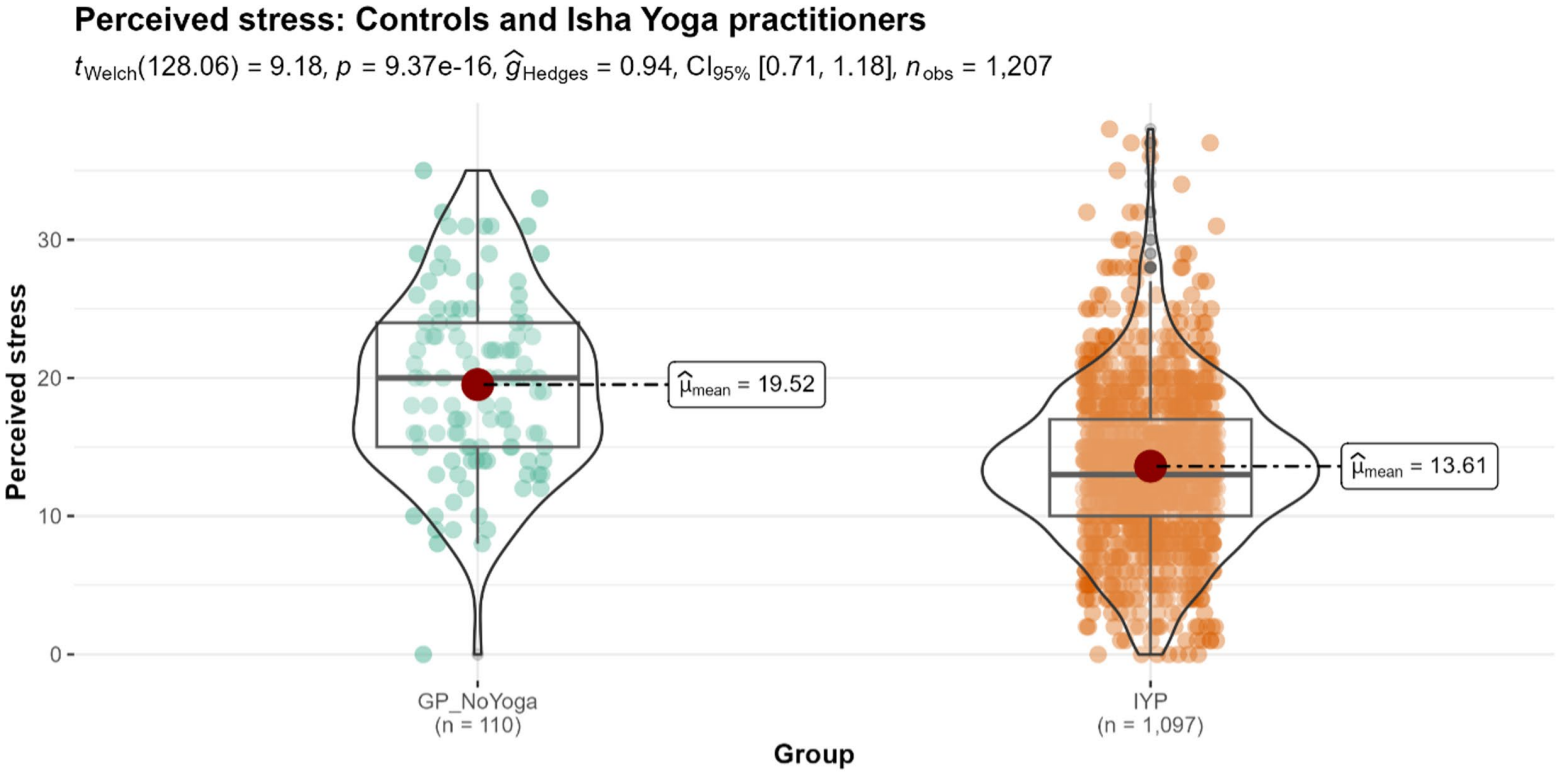
Differences in perceived stress score levels between controls and Isha meditators. GP_NoYoga, General population no Yoga (Controls); IYP, Isha Yoga practitioners.

The mean Perceived Stress Scale (PSS) score for the Isha Yoga practitioners is 13.61, which indicates low stress, whereas that of the controls is 19.52, which indicates moderate stress (*p* < 0.001) (Figure 1). The effect size was large.

#### 3.2.2 Mental distress

**Figure 2:**
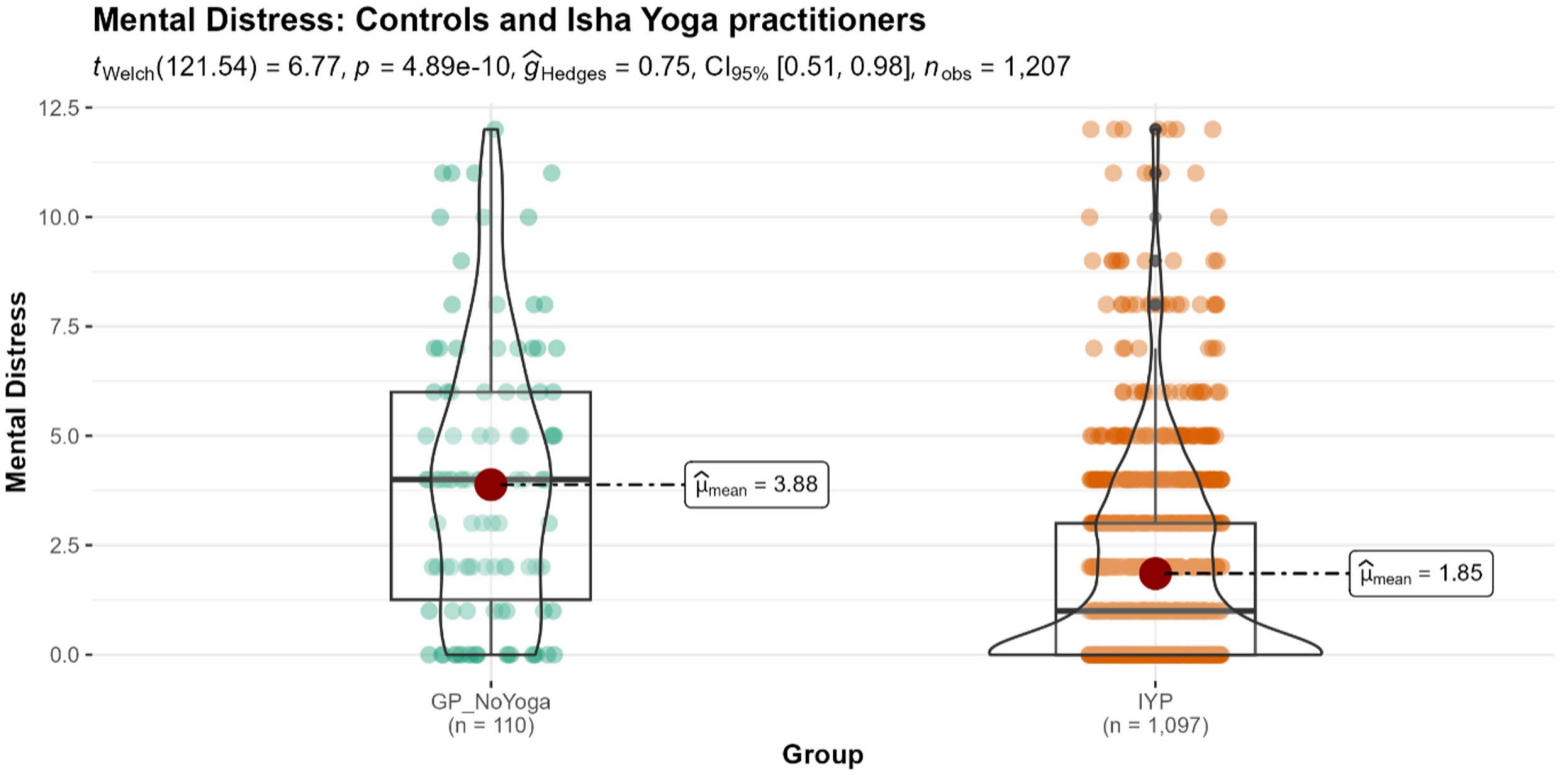
Differences in mental distress levels between controls and Isha meditators. GP_NoYoga, General population no Yoga; IYP, Isha Yoga practitioners.

The Isha Yoga practitioners (IYP) exhibited a mean mental distress score of 1.85, which is considered a normal value, whereas that of the control group is 3.88, which indicates moderate mental distress (*p* < 0.001) (Figure 2). This difference was associated with a medium effect size.

#### 3.2.3 Who well-being index

**Figure 3:**
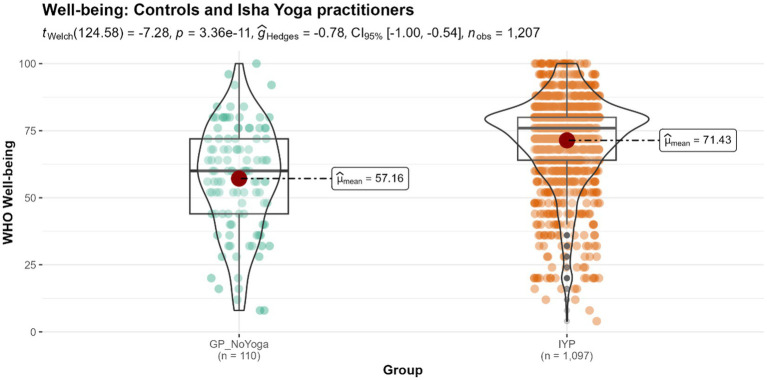
Differences in the levels of well-being between controls and Isha meditators. GP_NoYoga, General population no Yoga; IYP, Isha Yoga practitioners.

The mean WHO Well-being (WHO-5) score for Isha Yoga practitioners was 71.43, which was significantly higher compared to the control population with a score of 57.16 (*p* < 0.001) (Figure 3). We observed a medium effect size.

#### 3.2.4 SPANE balance

**Figure 4:**
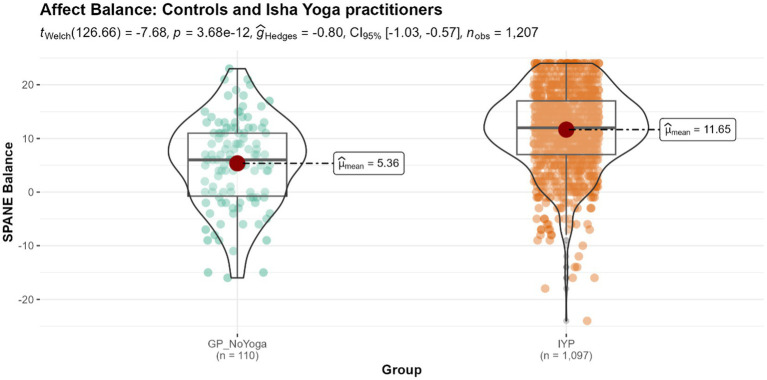
Differences in the levels of affect balance in controls and Isha meditators. GP_NoYoga, General population no Yoga; IYP, Isha Yoga practitioners.

Isha Yoga practitioners demonstrated significantly greater positive affective balance with a mean score of 11.65, compared to the control population with a mean score of 5.36 (*p* < 0.001) (Figure 4), with a large effect size.

Overall, we observed effect sizes ranging from medium to large. To contextualize these findings, meta-analyses have demonstrated that cognitive-behavioral therapy (CBT), a gold-standard treatment for mental health disorders such as anxiety and depression, typically yields effect sizes within the medium range (57–59).

### 3.3 Mental health outcomes: impact of expertise of Isha Yoga

**Figure 5:**
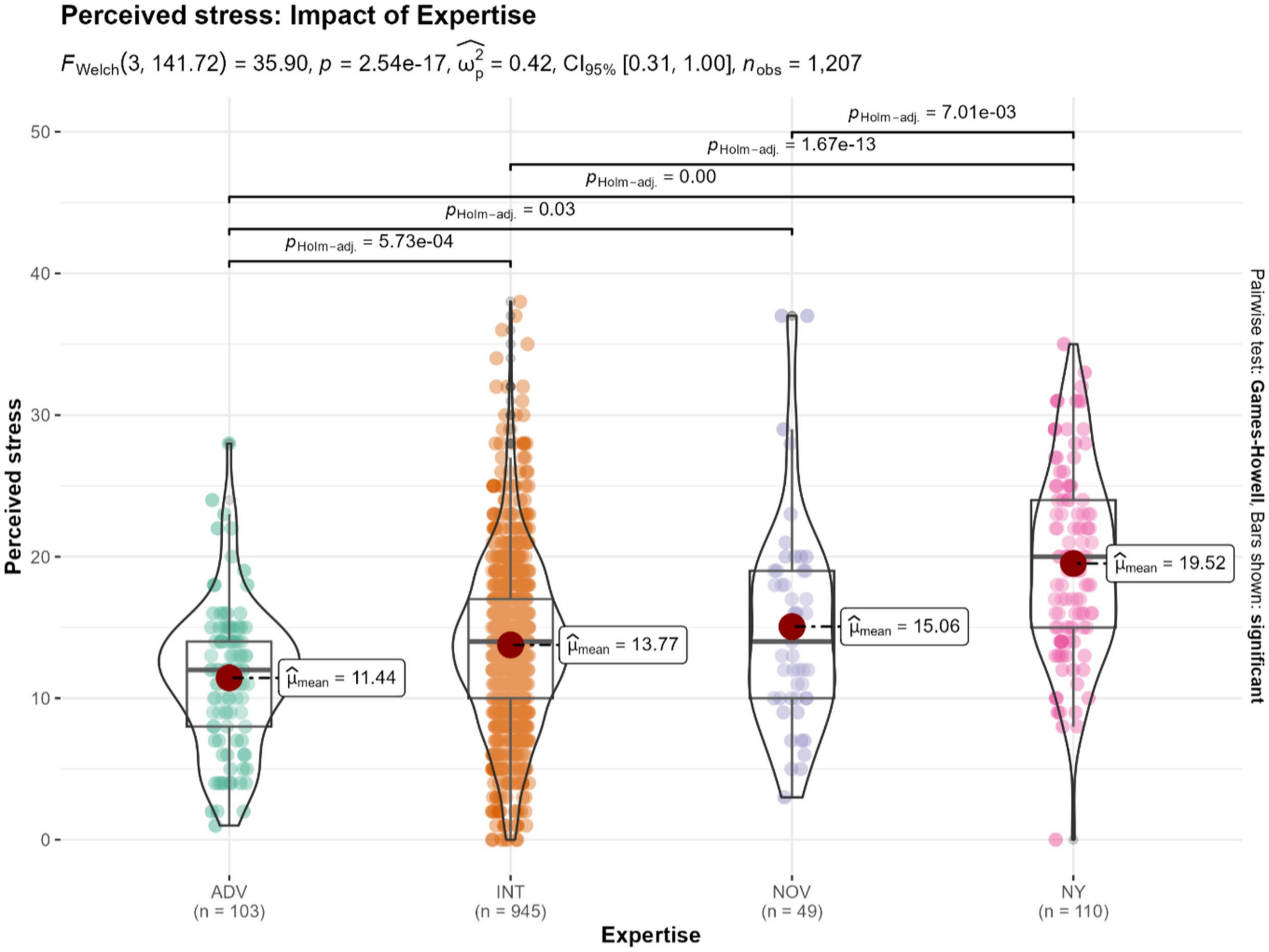
Differences in the levels of perceived stress, based on the impact of expertise among Isha Yoga practitioners. ADV, Advanced Isha Meditators (*n* = 103); INT, Intermediate Isha Meditators (*n* = 945); NOV: Novice Isha Meditators (*n* = 49); NY, No Yoga control group (*n* = 110).

The results of the one-way ANOVA analysis indicated a significant difference in mental health outcomes of perceived stress (Figure 5), mental distress, balance, and well-being (Supplementary Figures S1–S3) among the 1,207 subjects based on the expertise of the Isha Yoga practitioners. Specifically, advanced Isha practitioners exhibited the most significant differences from the control group across all four scales. Intermediate practitioners showed smaller differences compared to the control group, followed by novice practitioners. These findings suggest an association between the proficiency of practice of Isha Yoga and better mental health outcomes. Effect sizes were large.

### 3.4 Mental health outcomes: impact of regularity of Isha Yoga practice

**Figure 6:**
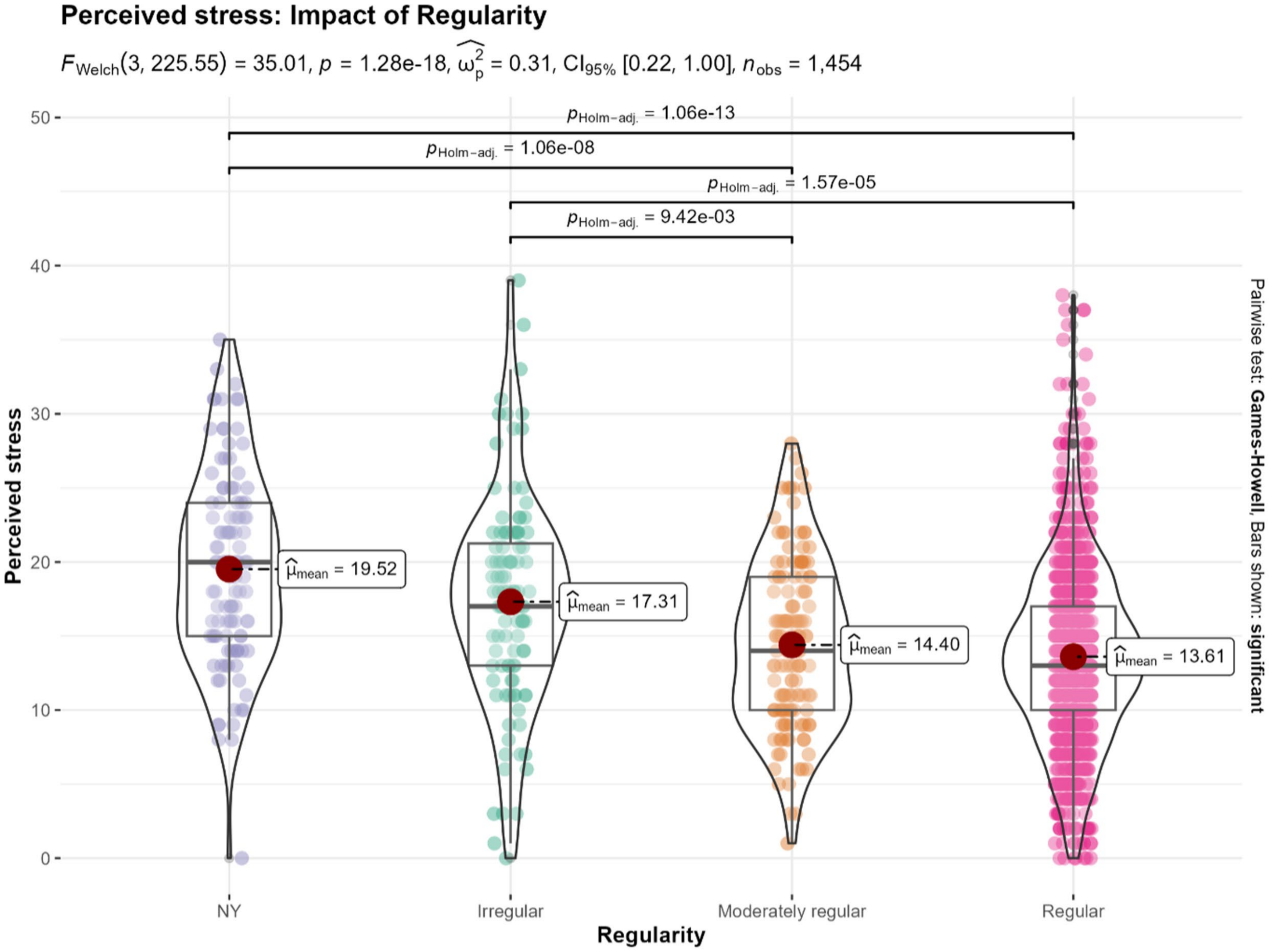
Differences in the levels perceived stress based on the impact of regularity of practice among Isha Yoga practitioners. NY: No Yoga control group (*n* = 110). Irregular: Less than 3 times of practice per week (*n* = 108). Moderately regular: 3–4 times of practice per week (*n* = 139). Regular: Minimum 5 times of practice per week (*n* = 1,097).

The analysis examining the impact of regularity of Isha Yoga practice revealed significant differences between the groups across all four scales (Figure 6; Supplementary Figures S4–S6). However, pairwise comparisons indicated no statistically significant differences between the irregular Isha Yoga practitioners (practicing less than 3 times per week) and the control group in any of the scales. Similarly, no statistically significant differences were observed between the moderately regular Isha practitioners (3–4 times of practice per week) and the regular Isha practitioners (minimum 5 times of practice per week) in any of the scales.

### 3.5 Mental health outcomes: by life-time hours of practice

**Figure 7:**
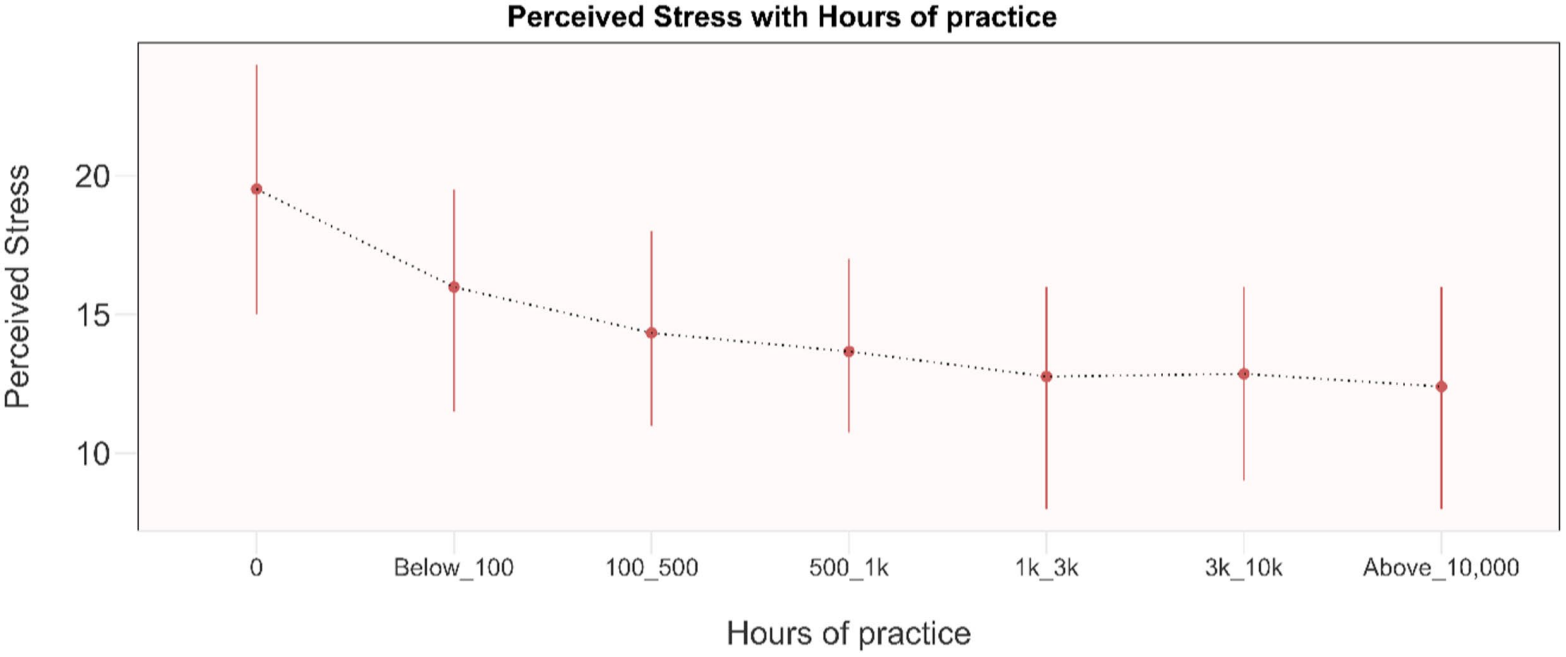
Dose–response relationship between perceived stress and total life-time hours of practice. Results are presented as mean and IQR. 0: No Yoga practice (*n* = 110). Below_100: Less than 100 life-time hours of Isha Yoga practice (*n* = 87). 100_500: 100 to 500 life-time hours of Isha Yoga practice (*n* = 289). 500_1k: 500 to 1,000 life-time hours of Isha Yoga practice (*n* = 228). 1k_3k: 1,000 to 3,000 life-time hours of Isha Yoga practice (*n* = 245). 3k_10k: 3,000 to 10,000 life-time hours of Isha Yoga practice (*n* = 158). Above_10,000: Greater than 10,000 life-time hours of Isha Yoga practice (*n* = 90).

**Figure 8:**
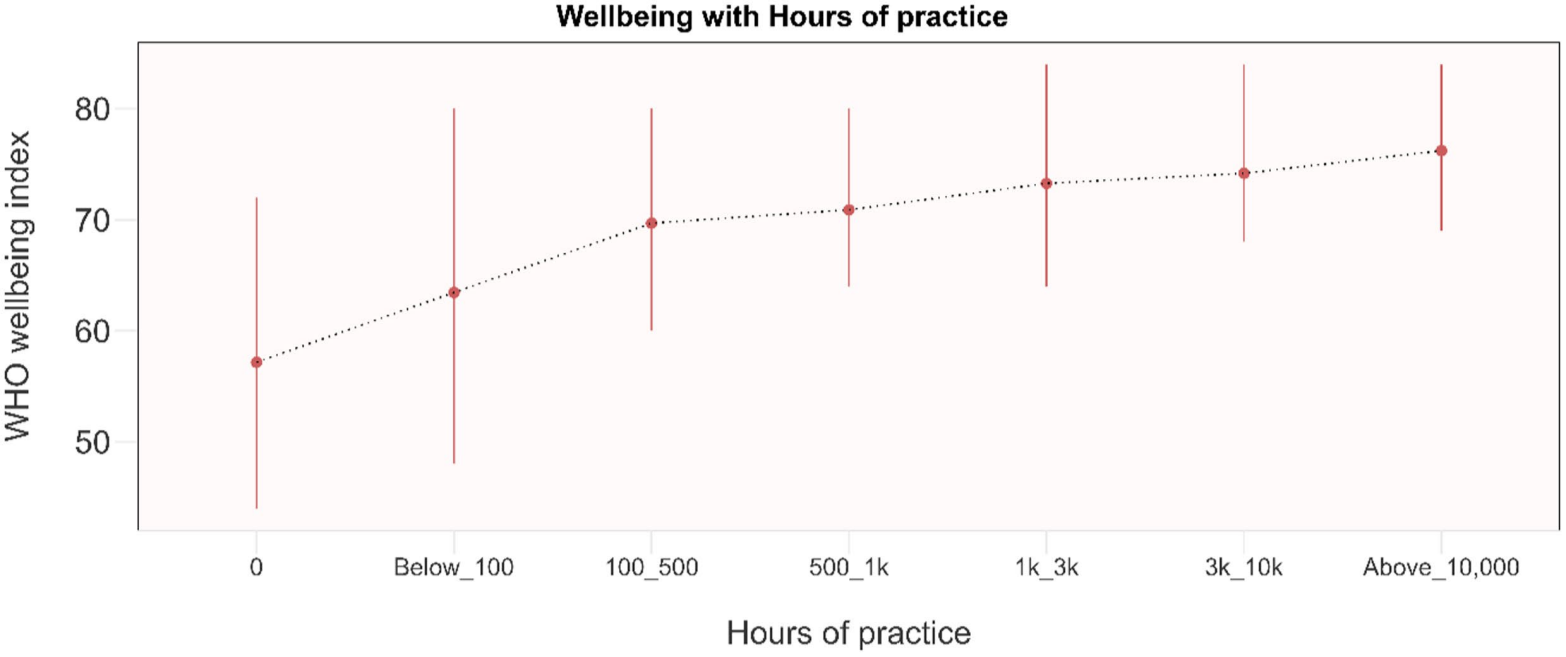
Dose–response relationship between WHO well-being and total life-time hours of practice. Results are presented as mean and IQR. 0: No Yoga practice (*n* = 110). Below_100: Less than 100 life-time hours of Isha Yoga practice (*n* = 87). 100_500: 100 to 500 life-time hours of Isha Yoga practice (*n* = 289). 500_1k: 500 to 1,000 life-time hours of Isha Yoga practice (*n* = 228). 1k_3k: 1,000 to 3,000 life-time hours of Isha Yoga practice (*n* = 245). 3k_10k: 3,000 to 10,000 life-time hours of Isha Yoga practice (*n* = 158). Above_10,000: Greater than 10,000 life-time hours of Isha Yoga practice (*n* = 90).

Most significant changes in mental health outcomes occurred within the initial 100 h of practice (Figures 7, 8; Supplementary Figures S7, S8). Scores showed a tendency to plateau after 500 h of practice, up to 3,000 h of practice. Results indicate an improvement in scores beyond 3,000 h of practice. These outcomes based on years of practice and practice time per day show similar changes (Supplementary Figures S9, S10).

### 3.6 Mental health outcomes: impact of Isha Yoga on health-care workers (HCW’s)

**Figure 9:**
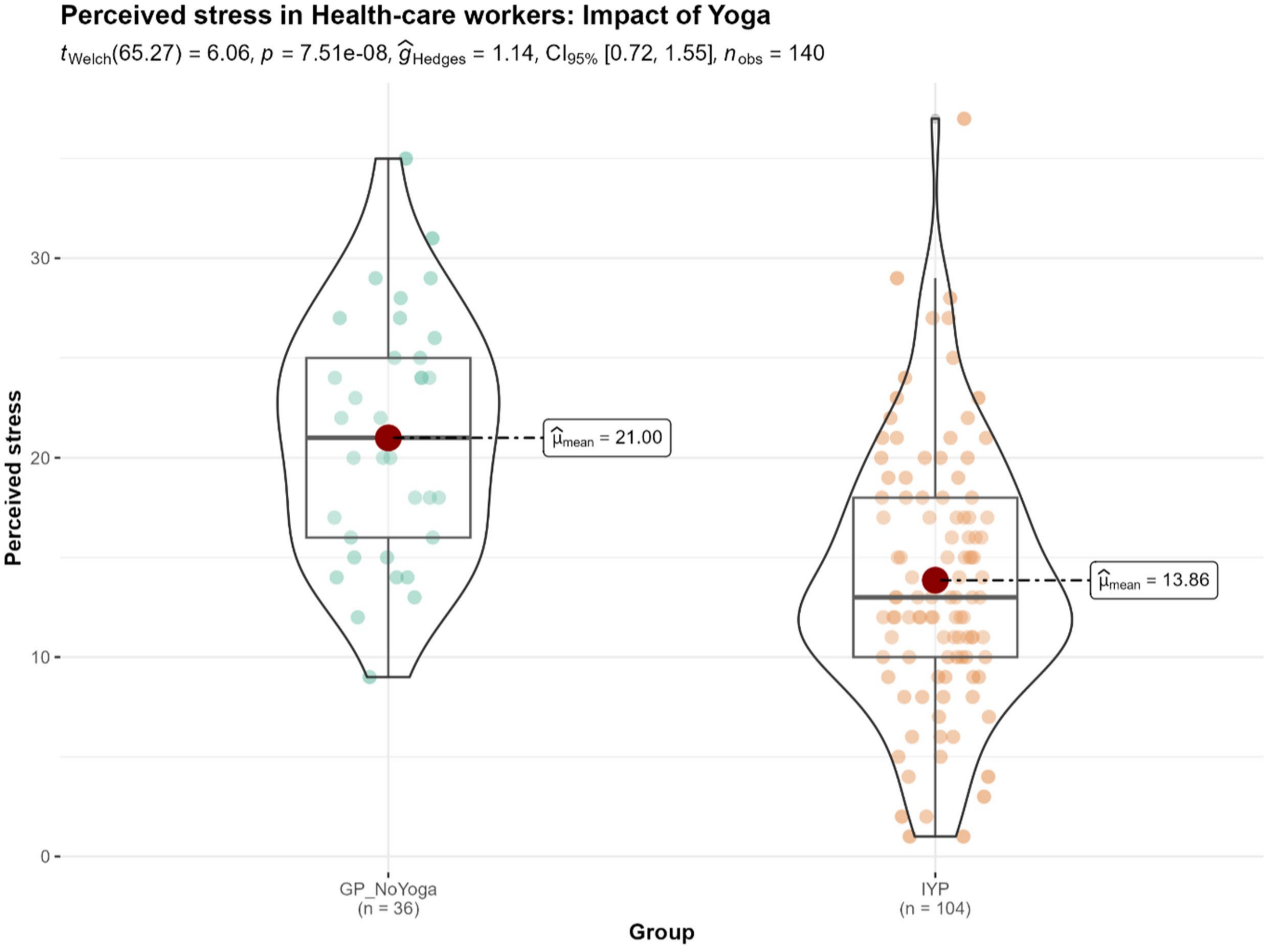
Differences in the levels of perceived stress in the Yoga-practicing health care workers (HCW’s) (*n* = 104) and no-Yoga HCW’s (*n* = 36). GP_NoYoga, General Population No Yoga; IYP, Isha Yoga practitioners.

Analyses indicated that there were statistically significant differences in all four scales (balance, mental distress, perceived stress, and well-being) between health care workers (HCW’s) who practiced Yoga and HCW’s who did not practice any form of Yoga (Figure 9; Supplementary Figure S11). Additionally, the effect sizes observed were medium (for mental distress and well-being) and large (for perceived stress and balance).

In addition to the outcomes discussed above, we also assessed sleep, diet, and physical activity among our survey respondents. The findings indicate that there were significant differences between the groups in both sleep quality (*p* < 0.001) (Supplementary Figure S12) and sleep quantity (*p* < 0.001) (Supplementary Figure S13). However, the effect size observed for both sleep quality and quantity was very small according to standard conventions (60). Our results also showed significant associations between Isha Yoga practises and the consumption of a pre-dominantly vegetarian diet (Supplementary Figure S14). There were no significant differences in physical activity levels between the groups (Supplementary Figure S15).

## 4 Discussion

Against the backdrop of a global increase in stress and stress-related disorders, including anxiety and depression (1, 3, 5, 13), our survey study, carried out during the COVID-19 pandemic, investigated the levels of perceived stress, mental distress, WHO well-being, and affect balance in a group of Isha Yoga practitioners and controls. The effect of proficiency and regularity in the Isha Yoga practise were also evaluated. Our findings revealed significant associations, with medium-to-large effect sizes, between the practise of Isha Yoga and better mental health outcomes. Proficiency played a key role, with advanced Isha Yoga practitioners showing the most favorable outcomes. The study underscored the importance of regular practice, as those non-compliant with Isha Yoga showed outcomes similar to controls. Further, a minimum 3–4 days of weekly practice showed significant differences with the control group. Importantly, our results suggest that even a relatively brief engagement in Isha Yoga, with fewer than 100 lifetime hours or less than a month of practice, is associated with better outcomes.

Traditional treatment approaches for stress-related disorders, such as anxiety and depression, often carry undesirable side effects (61, 62) and have been linked to the development of metabolic disorders (63). Compounding the challenge, approximately one third of individuals do not respond to conventional treatments, presenting a significant clinical hurdle (64, 65). At the same time, in India, there is a huge treatment gap of 80.4% (4). These factors underscore the necessity for preventive and integrative approaches in addressing these conditions. Meta-analyses have demonstrated that yoga practices may serve as valuable complementary treatment options for individuals dealing with anxiety and depression (66–68). Yogic breathing practises have also been shown to be effective for improving stress, anxiety, and depression, with small-to-medium mean effect sizes reported (69).

In this context, our findings of lower perceived stress and mental distress among Isha meditators compared to controls are promising. These results align with other studies on Isha meditators. For instance, a 10-min Isha Yoga practice, incorporating Yoga Namaskar and Nadi Shuddhi Pranayama, delivered online over 4 weeks, reduced perceived stress levels in US undergraduate students (25). Similarly, a 6-week practice of Shambhavi Mahamudra Kriya resulted in lower levels of perceived stress in a group attending a 4-day Inner Engineering retreat (31). Some studies on Isha Yoga have shown promising outcomes in reducing anxiety and depression. For instance, one study reported a statistically significant decrease in anxiety (effect size, *d* = 0.60) and depression (effect size, *d* = 0.48) among participants who attended an advanced 8-day Isha Samyama meditation retreat, with the effects sustained three months after the retreat. Those with higher baseline levels of anxiety and depression experienced greater reductions after the retreat (26). Similarly, another study observed a significant reduction in anxiety (by 23%, effect size = 0.60) and depression scores (by 26%, effect size = 0.31) among participants of a 4-day advanced Isha Yoga retreat (36). Despite these positive findings, further research on Isha Yoga, particularly employing randomized controlled trials with active comparators, is necessary to gain a more comprehensive understanding of the significance of Isha Yoga practices in reducing stress, anxiety, and depression.

While ample research exists to demonstrate the effectiveness of Mind and Body practices in mitigating negative outcomes, there is a relative scarcity of studies showcasing their impact on positive outcomes (70). In our survey, we found significant associations between Isha Yoga practises and higher levels of well-being and affect balance. Similar to ours, a study reported increased general well-being levels after six weeks of practice of Shambhavi Mahamudra (31). Furthermore, a randomized controlled trial (RCT) conducted with a waitlist control crossover assessed the effectiveness of 12 weeks of online Isha Upa Yoga intervention in US undergraduate students and found that the practise led to improved well-being scores in both the intervention and crossover control groups (25). Additionally, another study showed enhanced vitality, a sense of meaningful work, and workplace engagement among employees of a Fortune 500 company who participated in an Isha Inner Engineering Online program (32).

Similar improvements in positive outcomes have been found in studies on other practices like Sahaja Yoga and Sudarshan Kriya (71, 72).

Mind and Body practices (MBPs) like Isha Yoga likely bring about a reduction in negative outcomes and an increase in positive outcomes by enhancing overall self-regulation. Studies show that MBPs achieve this by reducing mind-wandering, increasing attention, and fostering present-moment awareness, enabling individuals to consciously respond to stress and uncertainty, even during challenging times like the COVID-19 pandemic (73–75). For instance, research has demonstrated increased attention in Isha Yoga practitioners after an advanced meditation retreat (35). Meditators also exhibit decreased default mode network (DMN) activity, a marker of mind-wandering (76). In Isha meditators, an advanced Isha Yoga program led to increased connectivity between the salience network (SN) and DMN, indicating reduced mind-wandering and heightened present-moment focus (77). Further, during deep meditation, there is an increase in theta and gamma EEG power, signifying heightened attention and awareness of the present moment. For instance, a study revealed that Isha Yoga practitioners experienced an increase in EEG gamma band power during Shoonya meditation (38). Certain brain regions crucial for attention and awareness, like the Pre-frontal Cortex, Insular Cortex, Anterior Cingulate Cortex, and Hippocampus, have been found to be altered in MBP practitioners (78, 79). Studies have shown that strengthened neural connections between pre-frontal and limbic regions in Yoga and meditation practitioners contribute to reduced stress and improved well-being (80–82).

Furthermore, Mind and Body practices (MBPs) exert beneficial effects in a bottom-up manner by impacting the parasympathetic and sympathetic nervous systems, reducing inflammation, and influencing the stress reactivity pathways (83). Isha Yoga, with its components of Hatha Yoga, Pranayama, and Kriyas, is believed to influence the mind in this manner. Studies have shown that Isha Yoga practitioners experience increased heart rate variability (HRV) and sympathovagal balance, indicating improved autonomic nervous system function (29, 84). Additionally, a study demonstrated a robust cortisol awakening response (CAR) in Isha Yoga practitioners following a 3-month retreat, suggesting better stress regulation (30). Likewise, another study reported 2-3-fold lower CRP levels (C-reactive protein, a marker of inflammation) in Isha meditators compared to controls, indicating lower systemic inflammation (26). Modulation of the Hypothalamic–Pituitary–Adrenal (HPA) axis and Sympathetic Adrenal Medullary (SAM) axis by MBPs creates a feedback loop to the brain, influencing mood and behavior, ultimately leading to reduced negative outcomes and enhanced positive ones. The two approaches, both top-down and bottom-up, work in synergy to provide a comprehensive framework for understanding how MBPs, such as Isha Yoga, have the potential to reduce negative outcomes and enhance positive ones, even in a high-stress population like health-care workers (HCWs). The interplay between the mind and body through these practices paves the way for greater self-regulation, emotional resilience, and a heightened sense of well-being.

Our study outcomes were influenced by the level of expertise in Yoga, with better results observed in individuals with more Isha Yoga experience, consistent with findings from other Yoga and meditation traditions (19, 20, 85, 86). Duration of practice and engagement in advanced Yoga techniques by experts likely contributed to these differences. Regularity of practice was also crucial, as irregular practitioners (less than 3 times per week) did not show significant differences compared to the control group, aligning with findings from other studies (23, 25, 31). We investigated the minimum amount of practice needed to yield benefits and found that practicing at least 3–4 times per week may be sufficient to decrease stress and promote well-being. The most significant changes tended to occur within the initial 100 life-time hours of practice with subsequent changes happening more gradually and plateauing. Even practicing Yoga for less than 30 min produced significant differences compared to not practicing at all. A study on Isha Yoga showed that reductions in perceived stress and improvements in general well-being only happened in those who were compliant with the practice (31). In addition, in a meta-analysis of mindfulness-based practices, researchers discovered a modest yet significant correlation between the amount of self-reported home practice and positive psychological outcomes (*r* = 0.26) (87). In contrast, another meta-analysis discovered no statistically significant relationship between the amount of daily practise and favorable outcomes (88). These contradictory findings necessitate further investigation. However, it is essential to recognize that the true purpose of Yoga goes beyond merely reducing stress and enhancing well-being. Yoga can have both state and trait effects on individuals (89, 90). These trait effects depend on consistent and regular practice, to the extent that one lives in that state of consciousness every moment. However, given that many people start practicing Yoga with the intention of reducing stress and improving well-being, our observations regarding these dose–response relationships could be encouraging. The results show that meaningful benefits can be experienced with relatively manageable commitments, serving as motivation to incorporate Yoga into daily routines and enhance overall well-being.

Considering factors such as diet, sleep, and physical activity is crucial when evaluating an individual’s health and well-being. In our study, we observed no statistically significant differences in physical activity levels between the control group and Isha meditators, suggesting that physical activity may not have directly contributed to the observed changes in mental health outcomes. However, we did find significant differences between the groups in terms of sleep and diet. Most of the Yoga practitioners shift to a predominantly vegetarian diet, as observed in our survey, following initiation into Yogic practises (26, 30). However, the link between a vegetarian diet and better mental health outcomes remains debatable, with conflicting evidence (91, 92). Future studies on Yoga should control for dietary factors to gain further insights. On the other hand, sleep has been consistently shown to influence mental health outcomes positively (93). It is plausible that practicing Yoga may have led to better sleep quality, thereby contributing to the observed improvements in mental well-being. Nevertheless, further research is necessary to gain a deeper understanding of this relationship.

### 4.1 Strengths and limitations

Internet surveys are prone to various biases, including the non-representative nature of the sample and the volunteer effect (self-selection of participants) (39, 40). A significant strength of our study is that we were able to precisely target our survey to the intended group. Moreover, our decision to focus exclusively on one Yoga tradition, Isha Yoga, contributed to the homogeneity of the Yoga type, thereby enhancing the study’s internal validity. Furthermore, the substantial number of survey respondents and a good participation rate of 71.53% further strengthen the study’s credibility. These factors collectively lead us to believe that the sample of our study is likely to be representative of Isha meditators in Karnataka, adding to the high external validity of our findings.

Another strength of our study lies in our adherence to appropriate guidelines for survey studies. Just as there are established guidelines for randomized trials (CONSORT) and systematic reviews (QUORUM), CHERRIES provide essential guidelines for conducting survey studies (41). By following these guidelines to the extent possible, we aimed to ensure the validity and credibility of our survey results, distinguishing our study from many others that may not adhere to such standards. Furthermore, our analysis was conducted in accordance with relevant guidelines (56, 60, 94) ensuring comprehensive and accurate statistical reporting. Additionally, we incorporated effect sizes for most of the observed outcomes, and the findings revealed medium-large effect sizes, further strengthening the significance of our results. These effect sizes suggest that the practice of Isha Yoga, regardless of its duration, is associated with a significant reduction in stress and improvement in well-being. However, it is important to acknowledge that our study’s cross-sectional design limits our ability to establish causality in the observed associations. To address this limitation and gain deeper insights, more longitudinal studies focusing on Isha meditators are warranted.

Lastly, the volunteer effect or the self-selection of participants may have biased our final sample. In addition, the selection of only English-speaking individuals in the study may have introduced a language and regional bias, potentially affecting the study’s generalizability to a broader population. Further, we relied on self-reported survey data to assess the outcomes, potentially introducing recall bias. To address this concern, future studies should aim to incorporate objective parameters alongside self-reported measures. Unfortunately, due to the constraints imposed by the COVID-19 pandemic, we were unable to include such objective assessments in our study. Nevertheless, future research should strive to overcome this limitation and integrate objective measurements to enhance the overall reliability of results.

## 5 Conclusion

Our study demonstrates significant associations between practice of Isha Yoga and better mental health outcomes, particularly during the COVID-19 pandemic. Isha Yoga practitioners showed lower levels of stress and mental distress, alongside higher well-being and balance, compared to controls. However, establishing a causal relationship is intricate, underscoring the need for longitudinal studies and randomized controlled trials. Expertise-related improvements were observed in these outcomes. The findings show a dose–response relationship between Isha Yoga practise and better outcomes. Importantly, our findings indicate that even a relatively brief engagement in Isha Yoga, with less than 100 lifetime hours of practice or less than a month of regular practice, is associated with notable improvements in outcomes. Additionally, the regularity of practice emerged as a crucial factor, as those not compliant with Isha Yoga practice demonstrated outcomes similar to controls.

Some of the strategies to improve compliance to Yoga practice are:

- Community engagement: Foster a sense of community through group sessions and community-based programs to provide social support and motivation.
- Incentives and rewards: Offer incentives such as discounts, rewards, or recognition to participants for consistent attendance and achievements.
- Technology integration: Develop and promote online resources, apps, or virtual classes to accommodate busy schedules and enhance accessibility.
- Personalized approaches: Tailor programs to individual needs and preferences. Offer one-on-one sessions or personalized plans for those with specific health concerns or limitations.

Future research should aim to overcome the limitations of the present study, including its cross-sectional design, reliance on subjective measurements, and the self-selection bias of participants. It is imperative to explore causal relationships and consider diverse samples from global datasets to account for cultural and social factors influencing various mental health outcomes. Additionally, it is crucial to emphasize that these practices should be regarded as a complementary intervention rather than a substitute for conventional mental health treatments. In conclusion, our findings highlight the potential of Isha Yoga practices as accessible and effective methods for enhancing well-being, supporting their integration into public health strategies.

Presented below are ten recommendations for policymakers and healthcare providers:

(a) Research and education

- Conduct research: Invest in scientific research to establish the effectiveness of yoga in promoting physical and mental health.
- Education programs: Develop educational programs to increase awareness about the benefits of yoga among healthcare professionals, policymakers, and the general public.

(b) Integration into healthcare systems

- Encourage healthcare providers to include yoga as part of treatment plans for various health conditions, such as stress, anxiety, depression, and chronic diseases.
- Training for healthcare professionals: Provide training for healthcare professionals to integrate yoga practices into their treatment approaches.

(c) Community-based programs

- Community centers and schools: Implement yoga programs in community centers, schools, and workplaces to make it accessible to diverse populations.
- Subsidies and incentives: Provide subsidies or incentives for organizations that incorporate yoga into their wellness programs.

(d) Accessibility and inclusivity

- Make yoga classes affordable or offer subsidies to ensure that individuals from all socioeconomic backgrounds can participate.
- Culturally sensitive approaches: Consider cultural diversity and tailor programs to be inclusive and respectful of different cultural backgrounds.

(e) Workplace wellness programs

- Incentives for employers: Provide incentives for employers to offer yoga classes as part of workplace wellness programs, recognizing the potential benefits for employee health and productivity
- Flexible scheduling: Encourage flexible scheduling to accommodate employees participating in yoga classes.

(f) Public awareness campaigns

- Promote benefits: Launch public awareness campaigns to highlight the physical and mental health benefits of yoga
- Online resources: Develop online resources, including videos and informational materials, to make yoga practices accessible to a broader audience.

(g) Collaboration with Yoga organizations

- Partnerships: Collaborate with yoga organizations and instructors to design and implement effective and evidence-based programs
- Certification standards: Establish certification standards for yoga instructors to ensure quality and safety.

(h) Policy support

- Insurance coverage: Advocate for insurance coverage of yoga classes when recommended by healthcare professionals for specific health conditions.
- Legal recognition: Recognize and support the legal standing of certified yoga instructors, ensuring their ability to contribute to public health initiatives.

(i) Research funding

- Allocate funding: Allocate funding for further research on the long-term health benefits of yoga and its potential role in preventive medicine

(j) Monitoring and evaluation

- Outcome evaluation: Implement systems to monitor and evaluate the outcomes of yoga programs in terms of public health impact, cost-effectiveness, and participant satisfaction.

## Data availability statement

The raw data supporting the conclusions of this article will be made available by the authors, without undue reservation.

## Ethics statement

The studies involving humans were approved by Nimhans Human Ethics Committee. The studies were conducted in accordance with the local legislation and institutional requirements. The participants provided their written informed consent to participate in this study.

## Author contributions

SaM: Conceptualization, Data curation, Formal analysis, Investigation, Methodology, Software, Visualization, Writing – original draft, Writing – review & editing. SeM: Methodology, Supervision, Writing – review & editing. JJ: Methodology, Supervision, Writing – review & editing. BK: Conceptualization, Methodology, Supervision, Writing – original draft, Writing – review & editing.
