# Supplementary material for: Practice and proficiency of Isha Yoga for better mental health outcomes: insights from a COVID-19 survey
Source: Front Public Health. 2024 Feb 2;12:1280859. doi: 10.3389/fpubh.2024.1280859 (PMC10869487; doi:10.3389/fpubh.2024.1280859)
Supplement: Supplementary file 1 [file Data_Sheet_1.docx]

**Supplementary information**:

Advertisement to participate in the survey -

The following information was provided to the meditators as part of the campaign:

“Namaskaram,

We invite you to participate in an online survey to study the impact of Isha Yoga practices on one's well-being.

This study is done by NIMHANS, Bangalore in collaboration with the Isha Foundation and inputs from the Sadhguru Centre for Conscious Planet at Harvard Medical School.

*Why this study?*

Mental health issues have risen dramatically since the Covid-19 outbreak. For policy-makers to know how effective Yoga is in boosting immunity, handling mental issues, and enhancing wellbeing, data is needed.

This study is a step in this direction.

*What do you need to do?*

Please note, there are two ways in which you can contribute to the survey.

- To participate in the survey, please click the button below
- Nominate and share this survey form with as many people from your friends or family members from Karnataka who don't practice any form of Yoga or meditation. This person should be of the same gender and fall in the same age range as yours (18 – 29, 30 – 44, 45 – 64, 65 or above).

Information provided in the survey would be kept confidential.

Pranam,

Isha volunteers”

**Supplementary Table 1 | Survey statistics for meditators are given below:**

| **Month** | **Mails sent to** | **Unique opens** | **Unique clicks** |
| --- | --- | --- | --- |
| August | 39,833 | 5977 (15%) | 690 |
| October | 40,682 | 9611 (24%) | 1200 |

Total unique opens: 15,588

Total unique clicks: 1890

Total responses: 1352

**Participation rate: 71.53% [(Total responses/Total unique clicks) *100]**

**Supplementary Table 2 | Practice characteristics of Isha Nominees**

| ***Practice of any form of Yoga, meditation or related practices*** | n (%) |
| --- | --- |
| Yes, currently practice | 111 (50.22%) |
| No, never practiced in life | 36 (16.28%) |
| Have a history of practice, lasted more than a month | 38 (17.19%) |
| Have a history of practice, lasted less than a month | 36 (16.28%) |

**Supplementary Table 3** **|** Categories of subjects and criteria used

| **Category** | **Criteria for selection** | **n** |
| --- | --- | --- |
| **Controls (CNT)** | Excluding all those who currently practice Yoga or any form of meditation | 110 |
| **Novice (NOV)**  **practioners** | Regular practice* AND less than 100 life-time hours of practice AND must have been initiated into Shambhavi Mahamudra and practicing it | 49 |
| **Intermediate (NTE) practitioners** | Regular practice* AND all others who do not belong to novice or advanced practitioner categories | 945 |
| **Advanced (ADV) practitioners** | Regular practice* AND more than 3000 life-time hours of practice AND must be practicing atleast one advanced Isha Yoga practice (like Shoonya, Shakti Chalana Kriya, or Samyama) | 103 |

*****Regular practice is defined as practicing Isha Yoga atleast 5 days per week.

**Supplementary Table 4 | Comparison of the Isha meditator group and Isha Yoga nominees**

| **Characteristics** | **Meditator group** | **Isha Yoga nominees** |
| --- | --- | --- |
| **Total sample (N)** | **1352** | **221** |
| ***Age-range (in years):*** | **n (percentage)** | **n (percentage)** |
| 18 – 29 | 287 (21.22%) | 86 (38.91%) |
| 30 - 44 | 714 (52.81%) | 86 (38.91%) |
| 45 - 64 | 307 (22.70%) | 42 (19%) |
| 65 years or above | 44 (3.25%) | 7 (3.16%) |
| ***Gender*** |  |  |
| Male | 797 (58.94%) | 104 (47.05%) |
| Female | 554 (40.97%) | 117 (52.94%) |
| Prefer not to say | 1 (0.07%) | 0 (0%) |
| ***Education*** |  |  |
| High school or some college | 49 (3.62%) | 22 (9.95%) |
| Bachelor’s degree or equivalent | 683 (50.51%) | 91 (41.17%) |
| Postgraduate degree | 601 (44.45%) | 104 (47.05%) |
| Prefer not to say | 19 (1.40%) | 4 (1.80%) |
| ***Healthcare worker*** |  |  |
| Yes | 124 (9.17%) | 62 (28.05%) |
| No | 1228 (90.82%) | 159 (71.94%) |
| ***Current medication use*** |  |  |
| Yes | 279 (20.63%) | 54 (24.43%) |
| No | 1073 (79.36%) | 167 (75.56%) |
| ***Health condition*** |  |  |
| Some medical condition | 453 (33.50%) | 60 (27.14%) |
| No medical condition | 899 (66.49%) | 161 (72.85%) |
| ***Alcohol consumption*** |  |  |
| Yes | 71 (5.25%) | 15 (6.78%) |
| Social drinker | 222 (16.42%) | 39 (17.64%) |
| No | 1059 (78.32%) | 167 (75.56%) |
| ***Smoking*** |  |  |
| Yes | 79 (5.84%) | 9 (4.07%) |
| No | 1273 (94.15%) | 212 (95.92%) |
| ***Substance use in the last 6 months*** |  |  |
| Yes | 35 (2.58%) | 7 (3.16%) |
| No | 1317 (97.41%) | 221 (96.83%) |
| ***Tested positive for Covid-19*** |  |  |
| Yes | 268 (19.82%) | 47 (21.26%) |
| No | 1084 (80.17%) | 174 (78.73%) |
| ***Vaccination status*** |  |  |
| Yes, both doses | 805 (59.54%) | 152 (68.77%) |
| Yes, single dose | 402 (29.73%) | 63 (28.50%) |
| No | 145 (10.72%) | 6 (2.71%) |

**Mental Health Outcomes: Impact of Expertise of Isha Yoga**


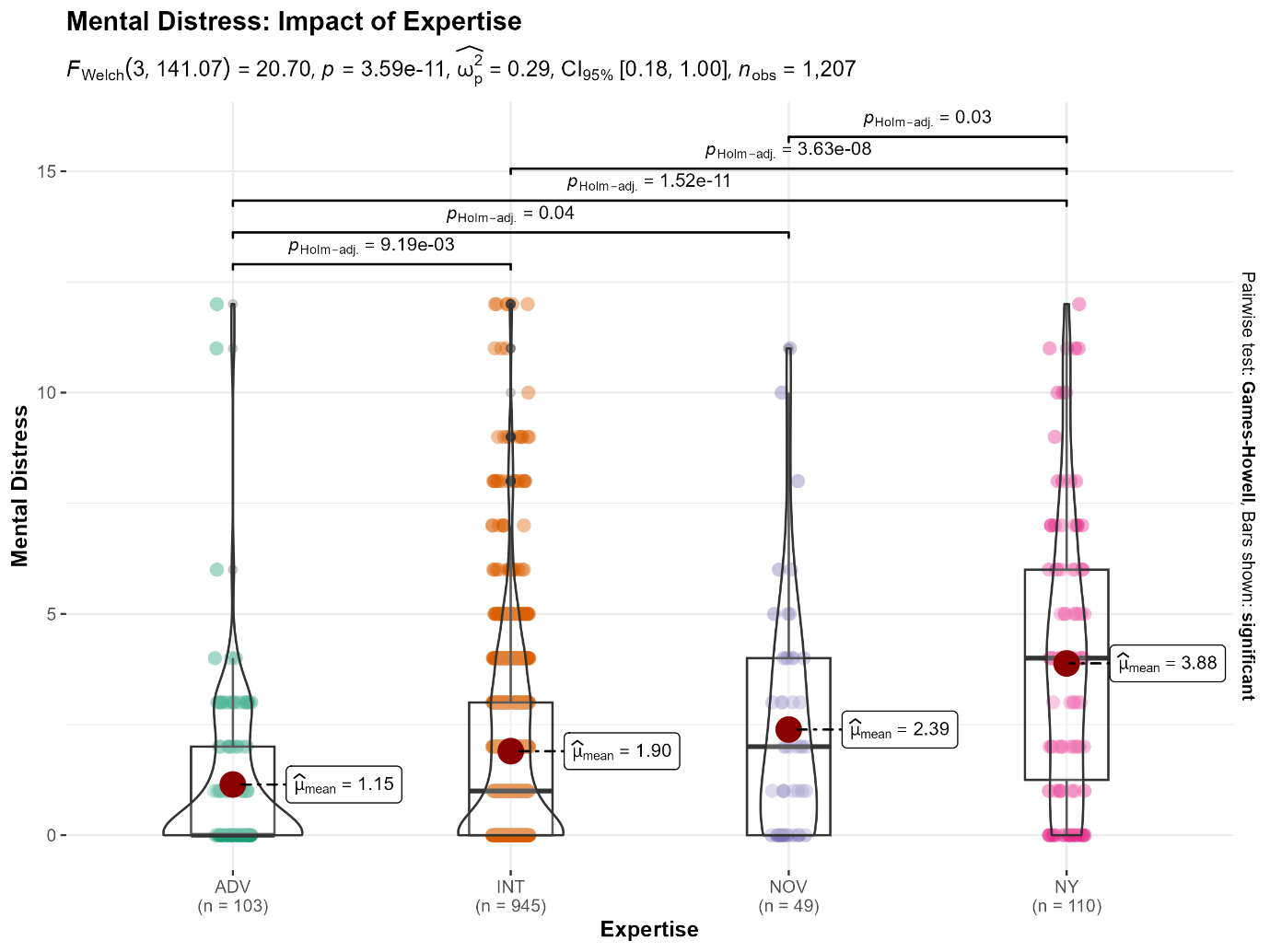


**Supplementary Figure 1:** Differences in the levels of mental distress based on the impact of expertise among Isha Yoga practitioners. ADV: Advanced Isha Meditators (n = 103). INT: Intermediate Isha Meditators (n = 945). NOV: Novice Isha Meditators (n = 49). NY: No Yoga control group (n = 110).


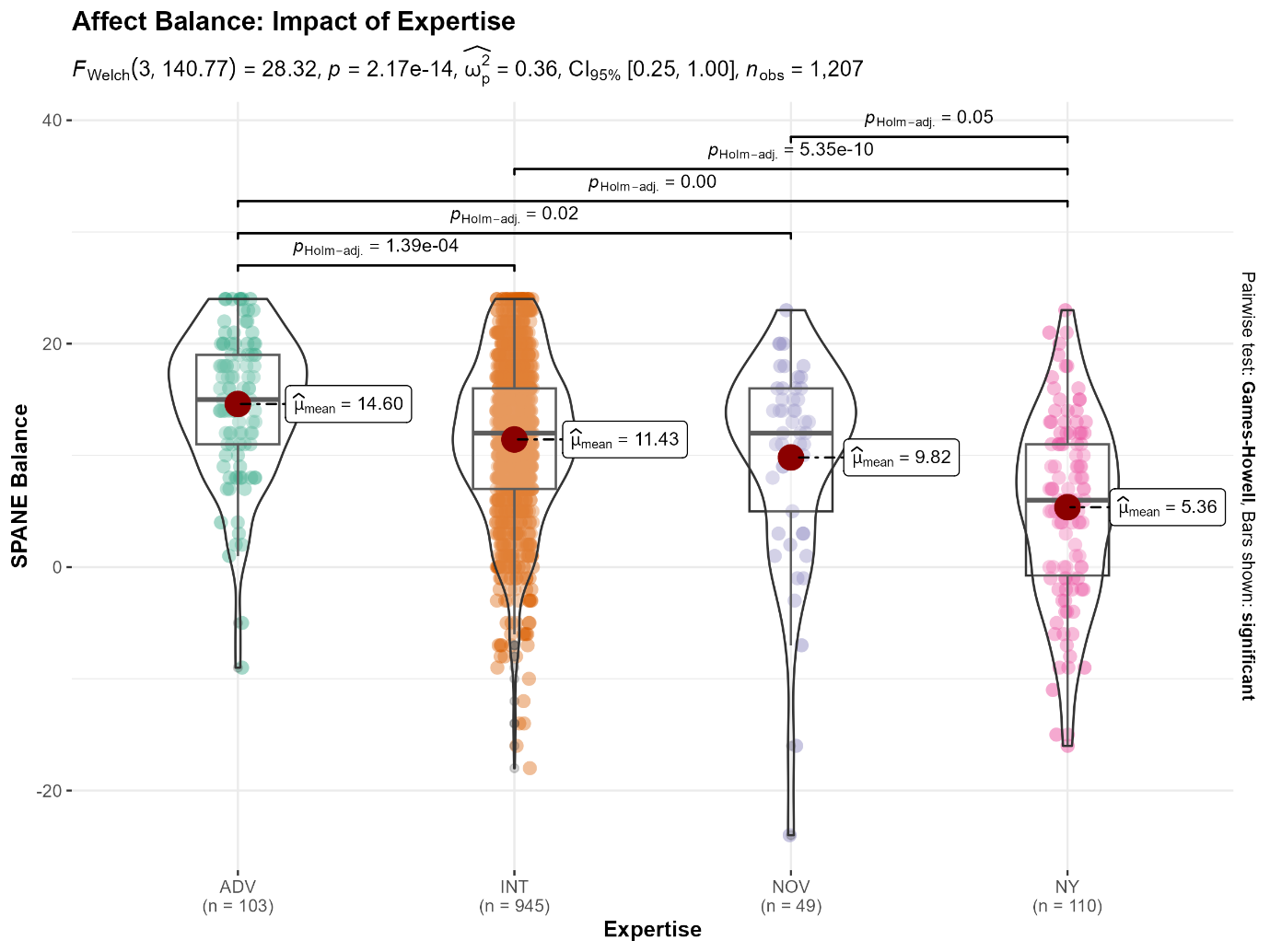


**Supplementary Figure 2:** Differences in the levels of affect balance based on the impact of expertise among Isha Yoga practitioners. ADV: Advanced Isha Meditators (n = 103). INT: Intermediate Isha Meditators (n = 945). NOV: Novice Isha Meditators (n = 49). NY: No Yoga control group (n = 110).


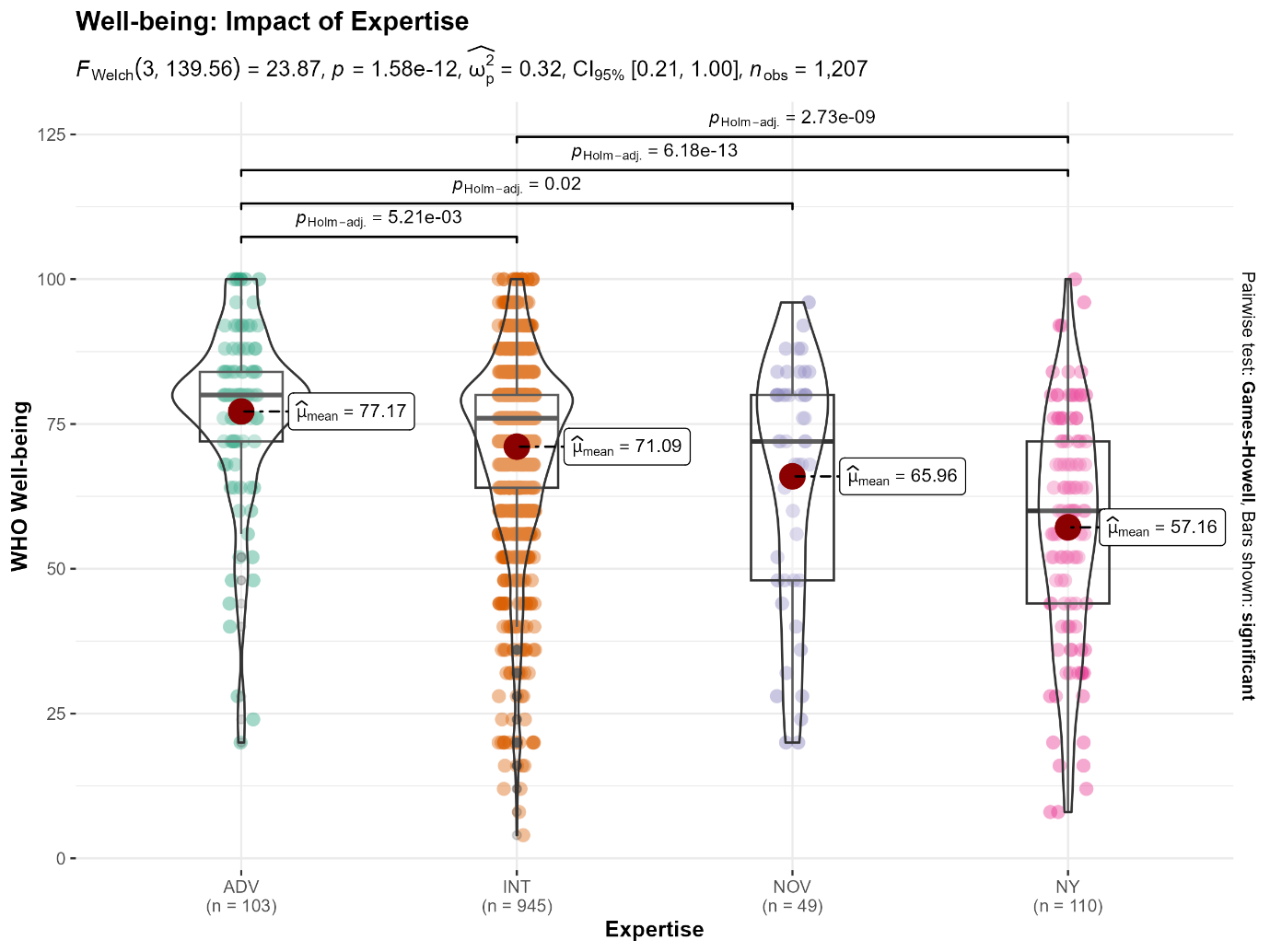


**Supplementary Figure 3:** Differences in the levels of well-being based on the impact of expertise among Isha Yoga practitioners. ADV: Advanced Isha Meditators (n = 103). INT: Intermediate Isha Meditators (n = 945). NOV: Novice Isha Meditators (n = 49). NY: No Yoga control group (n = 110).

**Mental Health Outcomes: Impact of Regularity of Isha Yoga practice**


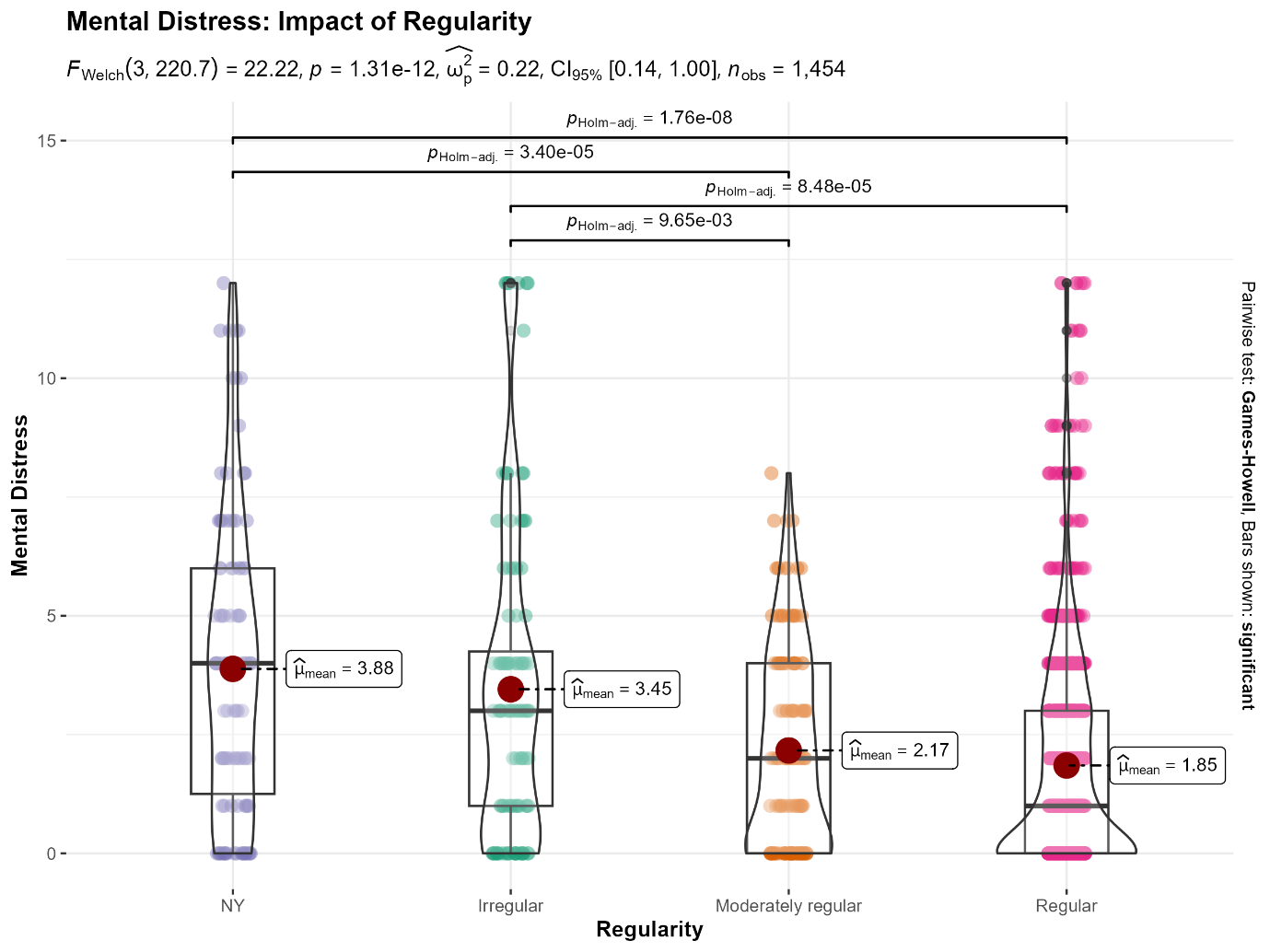


**Supplementary Figure 4:** Differences in the levels of mental distress based on the impact of regularity of practice among Isha Yoga practitioners. NY: No Yoga control group (n = 110). Irregular: Less than 3 times of practice per week (n = 108). Moderately regular: 3-4 times of practice per week (n = 139). Regular: Minimum 5 times of practice per week (n = 1,097).


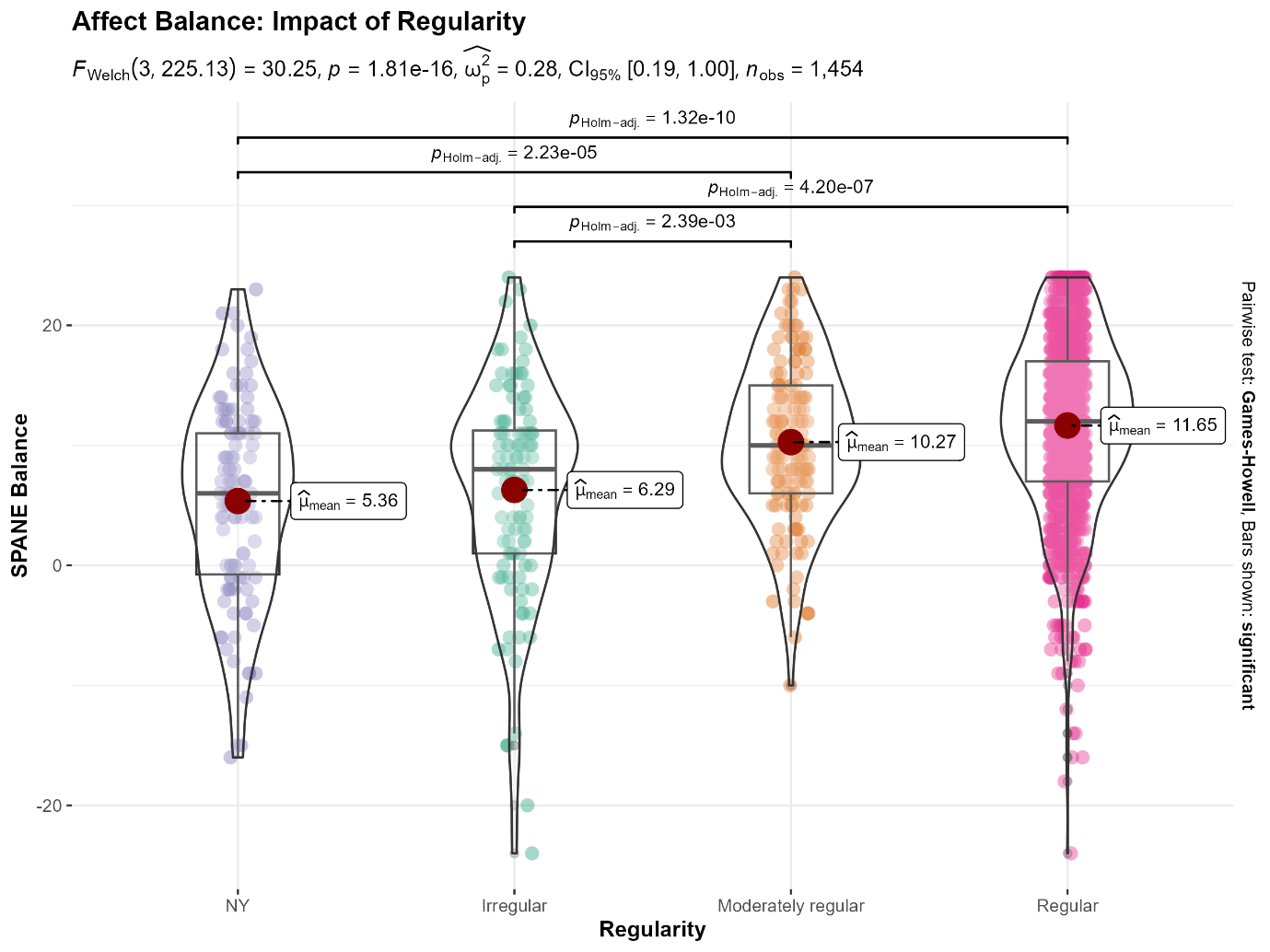


**Supplementary Figure 5:** Differences in the levels of affect balance based on the impact of regularity of practice among Isha Yoga practitioners. NY: No Yoga control group (n = 110). Irregular: Less than 3 times of practice per week (n = 108). Moderately regular: 3-4 times of practice per week (n = 139). Regular: Minimum 5 times of practice per week (n = 1,097).


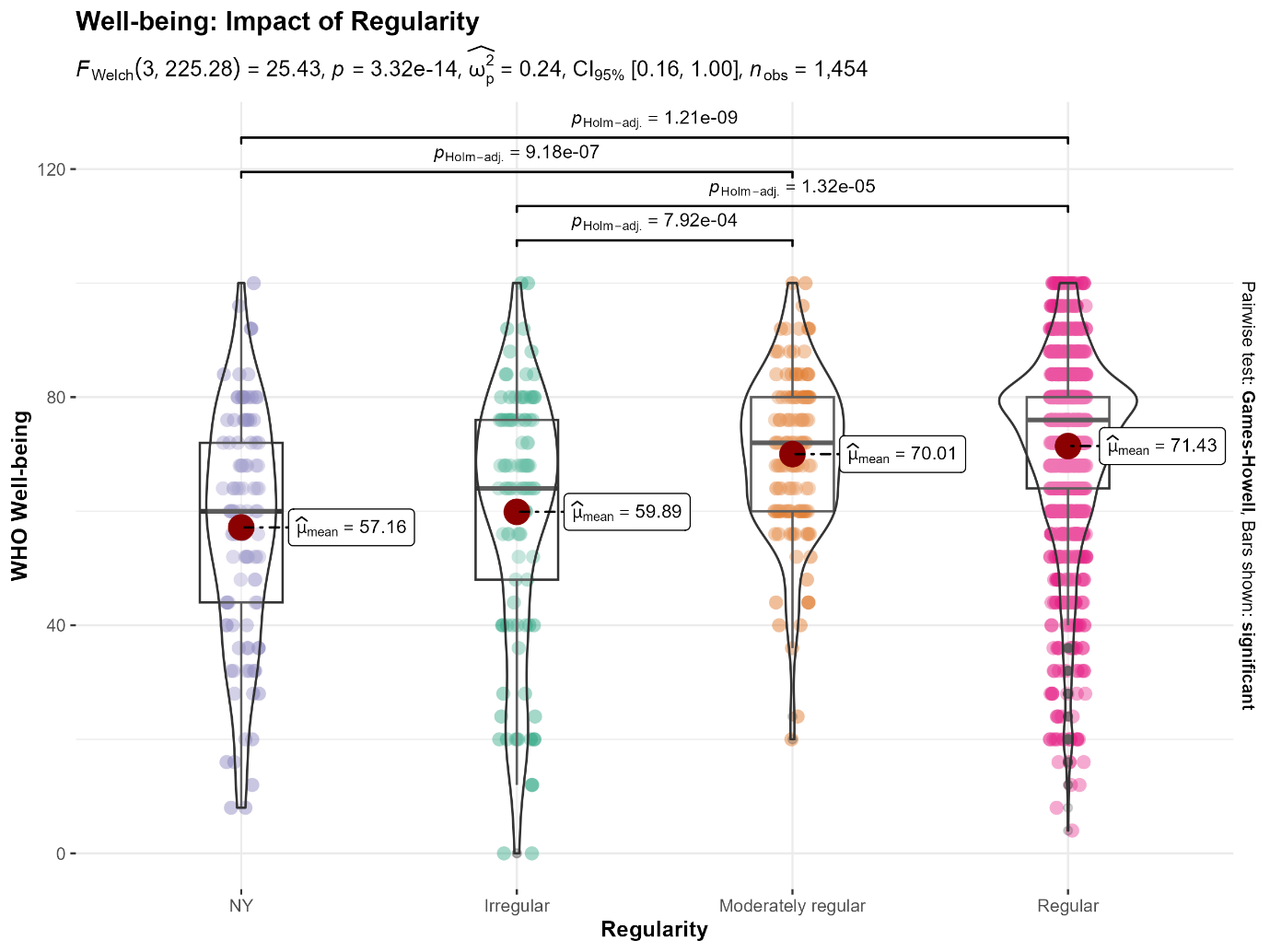


**Supplementary Figure 6:** Differences in the levels of WHO well-being based on the impact of regularity of practice among Isha Yoga practitioners. NY: No Yoga control group (n = 110). Irregular: Less than 3 times of practice per week (n = 108). Moderately regular: 3-4 times of practice per week (n = 139). Regular: Minimum 5 times of practice per week (n = 1,097).

**Mental Health Outcomes: By life-time hours of practice**


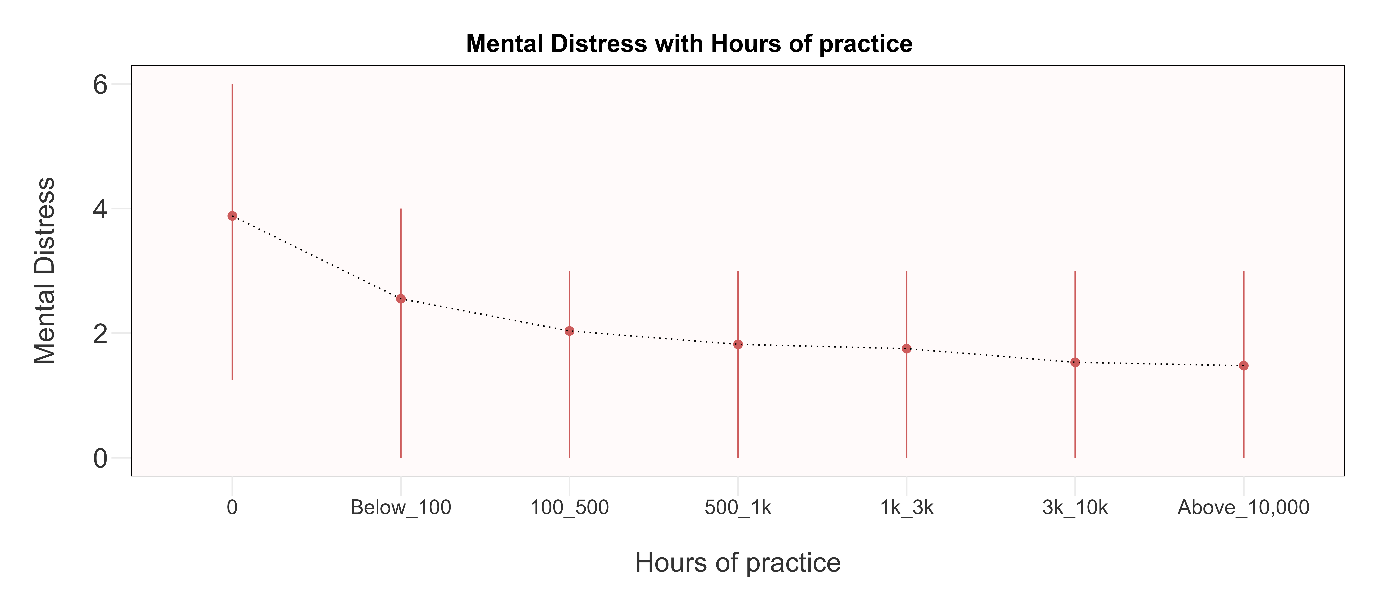


**Supplementary Figure 7:** Dose-response relationship between mental distress and total life-time hours of practice. Results are presented as mean and IQR. 0: No Yoga practice (n = 110). Below_100: Less than 100 life-time hours of Isha Yoga practice (n = 87). 100_500: 100 to 500 life-time hours of Isha Yoga practice (n = 289). 500_1k: 500 to 1000 life-time hours of Isha Yoga practice (n = 228). 1k_3k: 1000 to 3000 life-time hours of Isha Yoga practice (n = 245). 3k_10k: 3000 to 10,000 life-time hours of Isha Yoga practice (n = 158). Above_10,000: Greater than 10,000 life-time hours of Isha Yoga practice (n = 90).


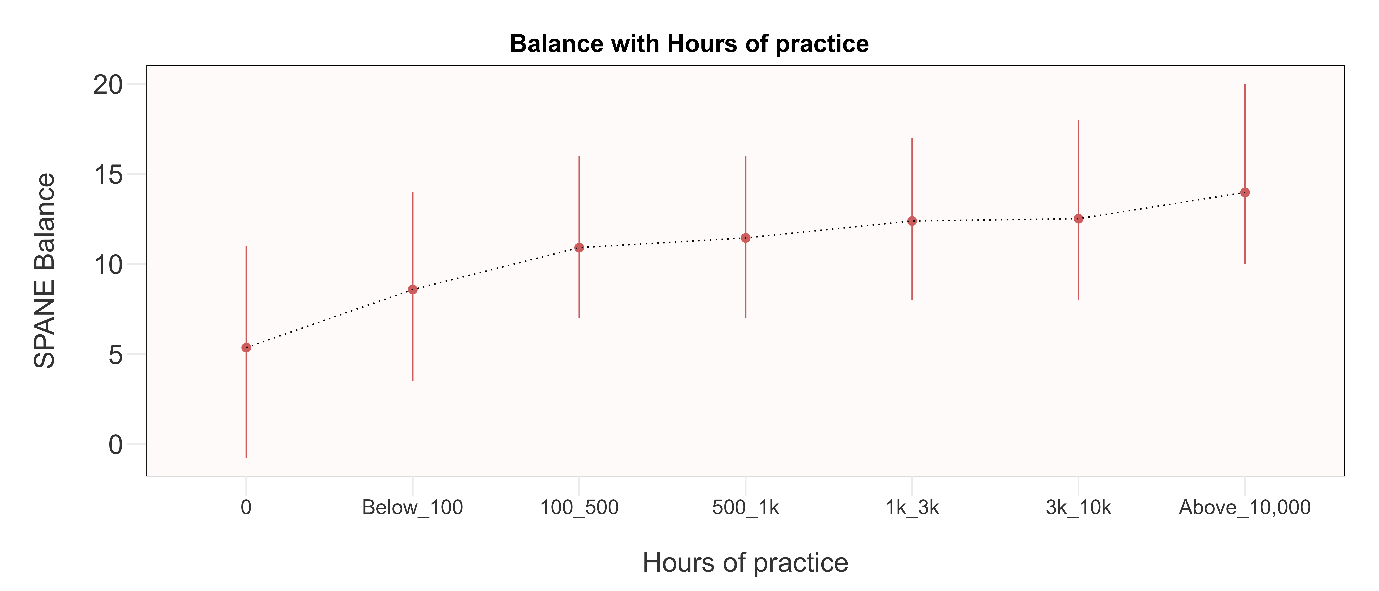


**Supplementary Figure 8:** Dose-response relationship between affect balance and total life-time hours of practice. Results are presented as mean and IQR. 0: No Yoga practice (n = 110). Below_100: Less than 100 life-time hours of Isha Yoga practice (n = 87). 100_500: 100 to 500 life-time hours of Isha Yoga practice (n = 289). 500_1k: 500 to 1000 life-time hours of Isha Yoga practice (n = 228). 1k_3k: 1000 to 3000 life-time hours of Isha Yoga practice (n = 245). 3k_10k: 3000 to 10,000 life-time hours of Isha Yoga practice (n = 158). Above_10,000: Greater than 10,000 life-time hours of Isha Yoga practice (n = 90).

**Mental Health Outcomes: By years of practice**

Results demonstrated that the most significant improvements in mental distress, perceived stress, and balance occurred during the initial three months of practice. Within this period, mean scores for mental distress reduced by 2 units, and perceived stress showed a linear decrease from 20 units to 15 units. Similarly, scores for balance linearly increased from 5 to 10. Following this initial phase, the scores stabilized for mental distress and perceived stress for up to two years, with further reductions observed after that period. For balance, slight changes were noted in scores after the initial three months of practice. Regarding WHO well-being, the most substantial changes were observed in less than one month of practice, with a notable improvement of 10 units in mean scores. Subsequently, there were gradual increases in well-being scores with increasing years of practice. Overall, the findings suggest that the initial months of practice yield maximal improvements in mental distress, perceived stress, balance, and well-being, with continued positive effects over time for most measures.


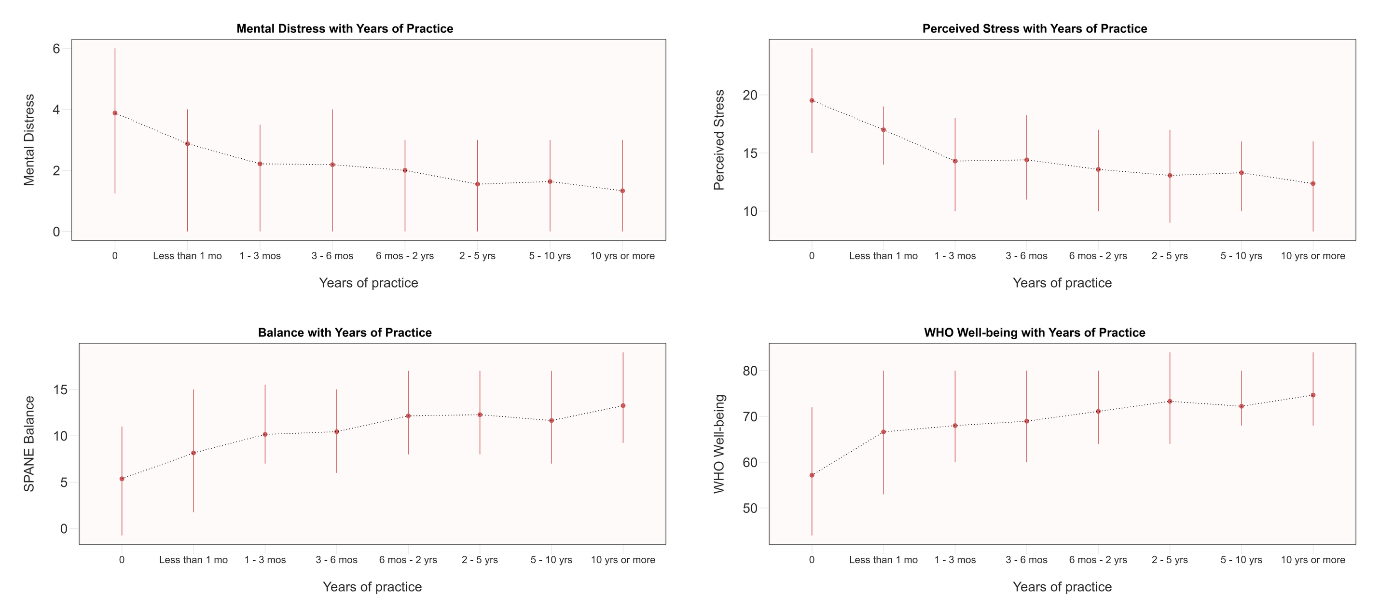


**Supplementary Figure 9:** Dose-response relationship between different mental health outcome measures and years of practice of Isha Yoga. Results are presented as mean and IQR. 0: No Yoga practice (n = 110). Less than 1 mo: Less than 1 month of practice of Isha Yoga (n = 32). 1 – 3 mos: 1 – 3 months of practice of Isha Yoga (n = 111). 3 – 6 mos: 3 – 6 months of practice of Isha Yoga (n = 148). 6 mos – 2 yrs: 6 months to two years of practice of Isha Yoga (n = 264). 2 – 5 yrs: Two to five years of practice of Isha Yoga (n = 386). 5 – 10 yrs: Five to ten years of practice of Isha Yoga (n = 102). 10 yrs or more: More than 10 years of practice of Isha Yoga (n = 54).

**Mental Health Outcomes: By practice time per day**

Results revealed a consistent linear decrease in scores (for mental distress and perceived stress) with increasing practice-time per day. Those practicing for less than 30 minutes per day showed significant differences compared to those who did not practice any Yoga at all. Isha Yoga practitioners engaging in 4 hours or more practice per day experienced a notable decrease of more than 2 units in mental distress scores and more than 7 units in perceived stress scores. Regarding balance, scores demonstrated linear increases with increasing practice-time. Participants practicing for less than 30 minutes exhibited an increase of more than 2 units in their balance scores. For well-being, scores also exhibited a linear increase with more extended practice-time. Notably, even those practicing for less than 30 minutes showed a significant improvement in scores. Overall, the results show that even practicing Yoga for less than 30 minutes daily can lead to a considerable improvement in mental distress, perceived stress, balance, and well-being scores. However, engaging in longer practice sessions, especially 4 hours or more, results in even more pronounced positive effects.


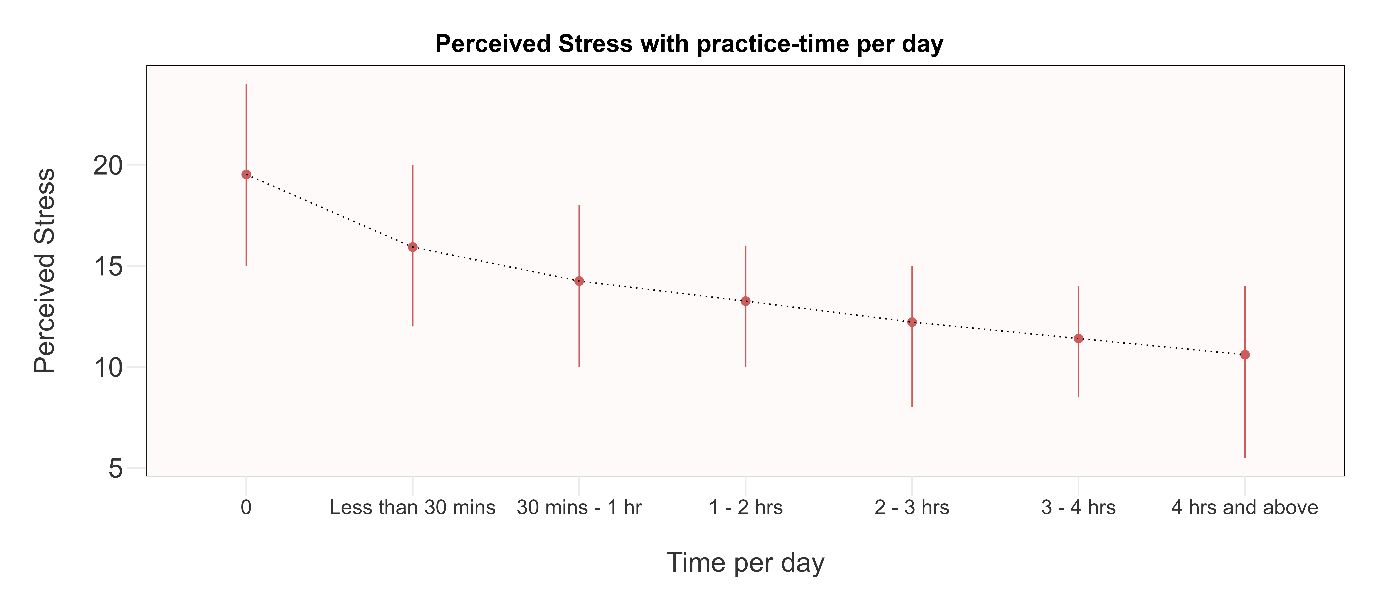

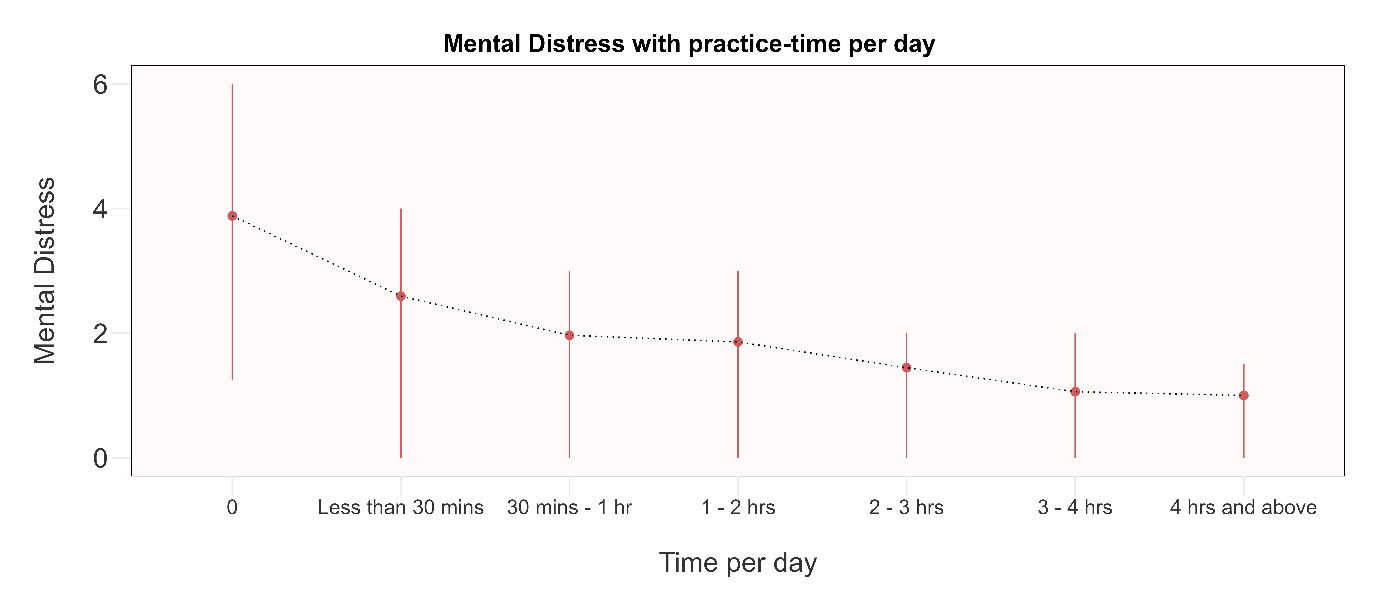

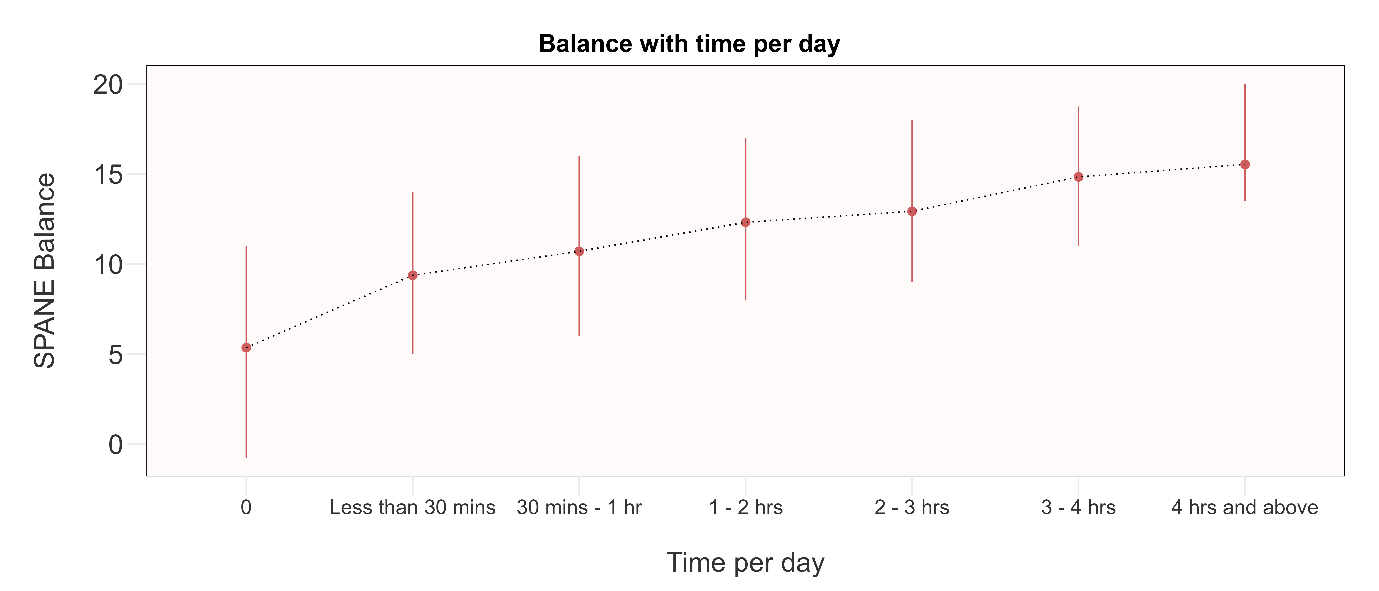


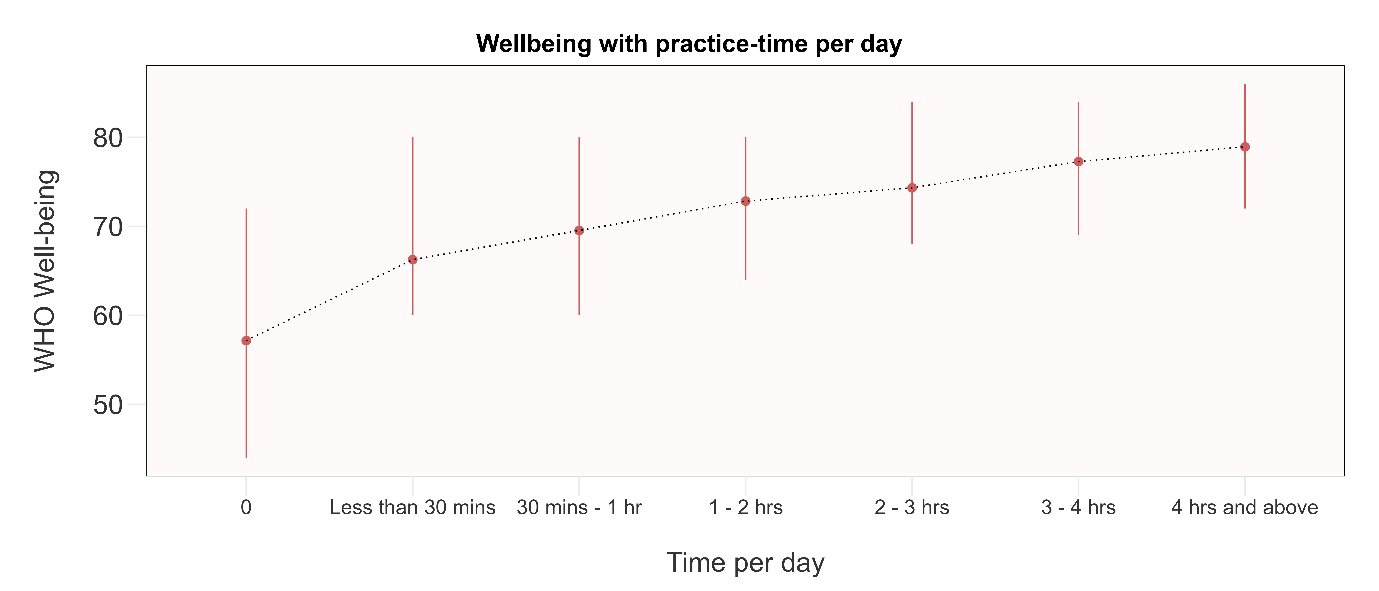


**Supplementary figure 10:** Dose-response relationship between different mental health outcome measures and practice time per day of Isha Yoga. Results are presented as mean and IQR. 0: No Yoga practice (n = 110). Less than 30 mins of Isha Yoga practice (n = 69). 30 mins to 1 hour of Isha Yoga practice (n = 491). 1 to 2 hours of Isha Yoga practice (n = 326). 2 to 3 hours of Isha Yoga practice (n = 146). 3 to 4 hours of Isha Yoga practice (n = 50). 4 hrs and above of Isha Yoga practice (n = 15).

**Mental Health Outcomes: Impact of Isha Yoga on Health-care workers (HCW’s)**

**
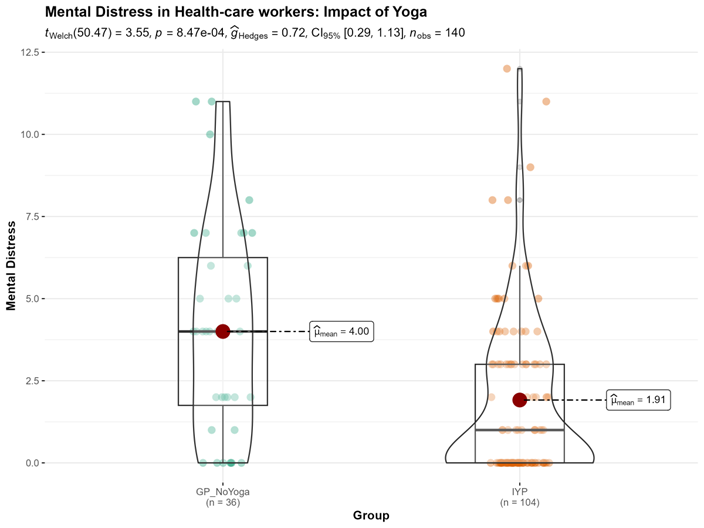
**


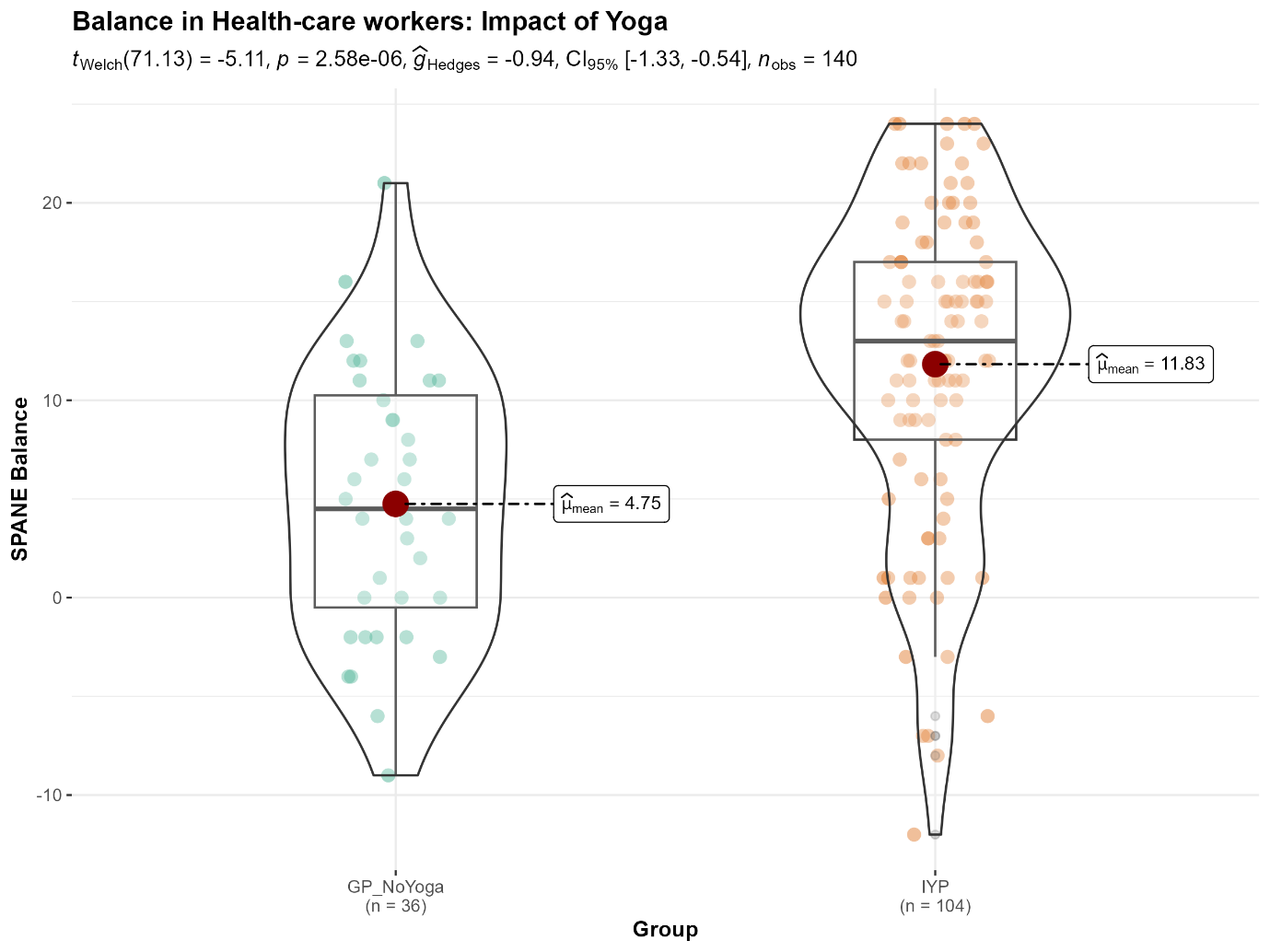

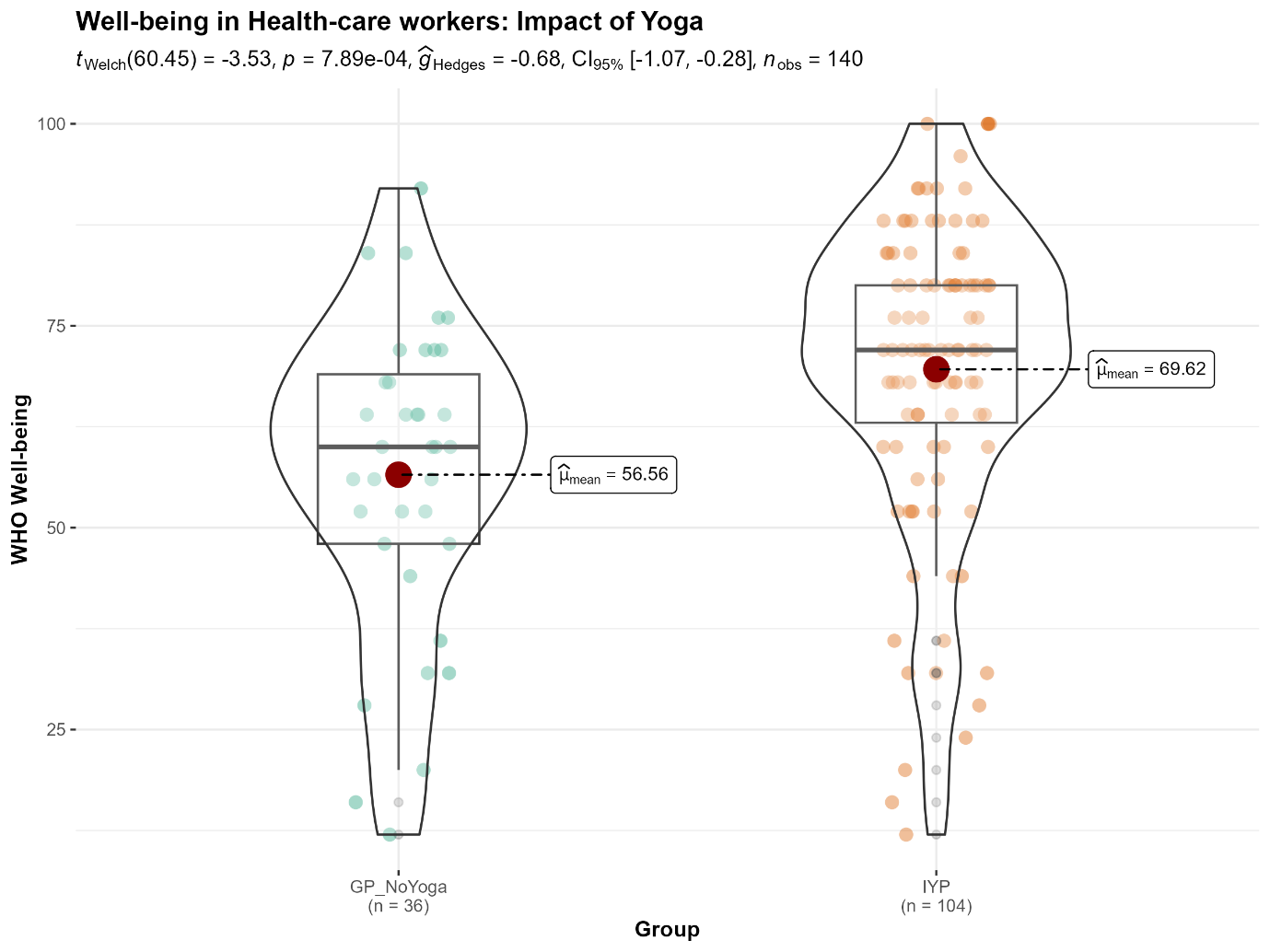


**Supplementary figure 11:** Differences in the levels of mental distress, balance, and well-being in the Yoga-practicing health care workers (HCW’s) (n = 104) and no-Yoga HCW’s (n = 36). GP_NoYoga – General Population No Yoga. IYP – Isha Yoga practitioners.

**Sleep quantity and quality**

The findings indicate that there were significant differences between the groups in both sleep quality (p = 1.79e-04) (Supplementary Figure 12) and sleep quantity (Supplementary Figure 13). However, the effect size observed for both sleep quality and quantity was very small according to standard conventions (59).


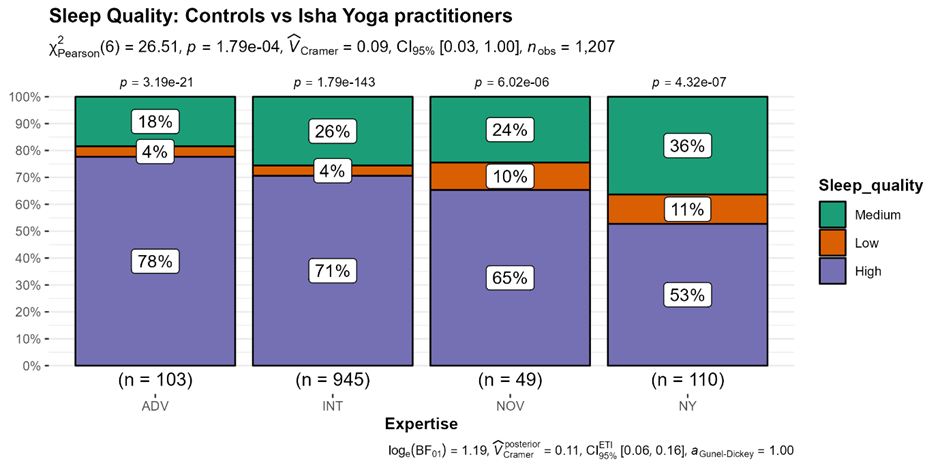


**Supplementary figure 12:** Differences in sleep quality between the Isha meditators and controls. ADV: Advanced Isha practitioners. INT: Intermediate Isha practitioners. NOV: Novice Isha practitioners. NY: No Yoga control group.


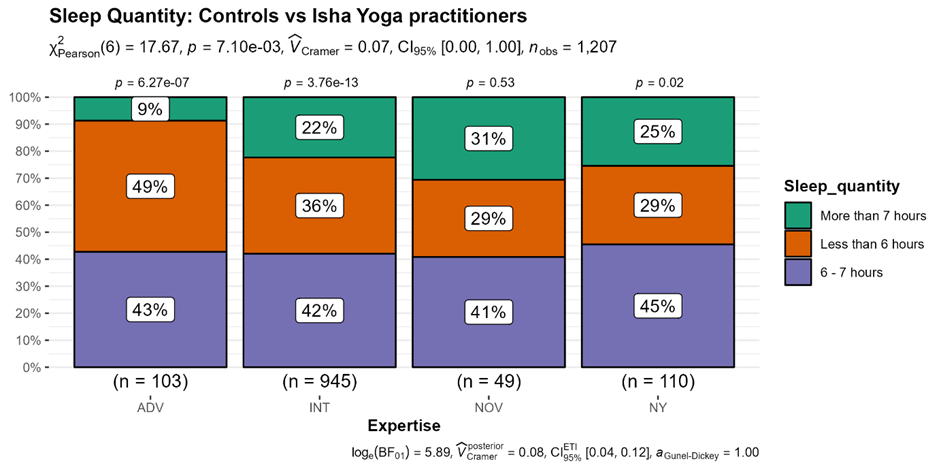


**Supplementary figure 13:** Differences in sleep quantity between the Isha meditators and controls. ADV: Advanced Isha practitioners. INT: Intermediate Isha practitioners. NOV: Novice Isha practitioners. NY: No Yoga control group.

**Diet**

The findings indicated significant differences in diet between the groups (p = 2.51e^-10^) (Supplementary Figure 14). The effect size was small according to Funder's conventions (39). Among the control group, 70% consumed a non-vegetarian diet, whereas among the advanced meditators, only 30% did. As practitioners advanced and deepened their practices, the results suggested a progressive shift towards a vegetarian diet.


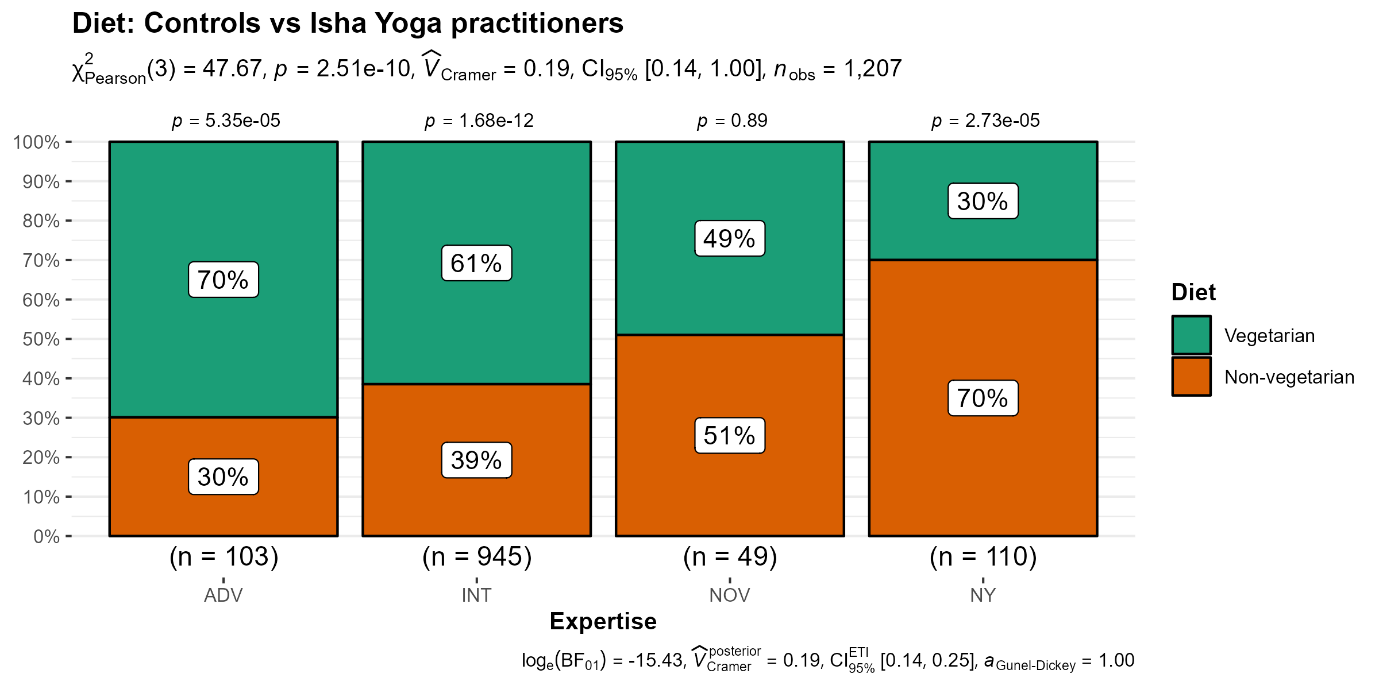


**Supplementary figure 14:** Differences in diet between the Isha meditators and controls. Non-vegetarian includes consumption of eggs. ADV: Advanced Isha practitioners. INT: Intermediate Isha practitioners. NOV: Novice Isha practitioners. NY: No Yoga control group.

**Physical activity**

The findings revealed no significant differences in physical activity levels between the groups (p = 0.10) (Supplementary Figure 15).


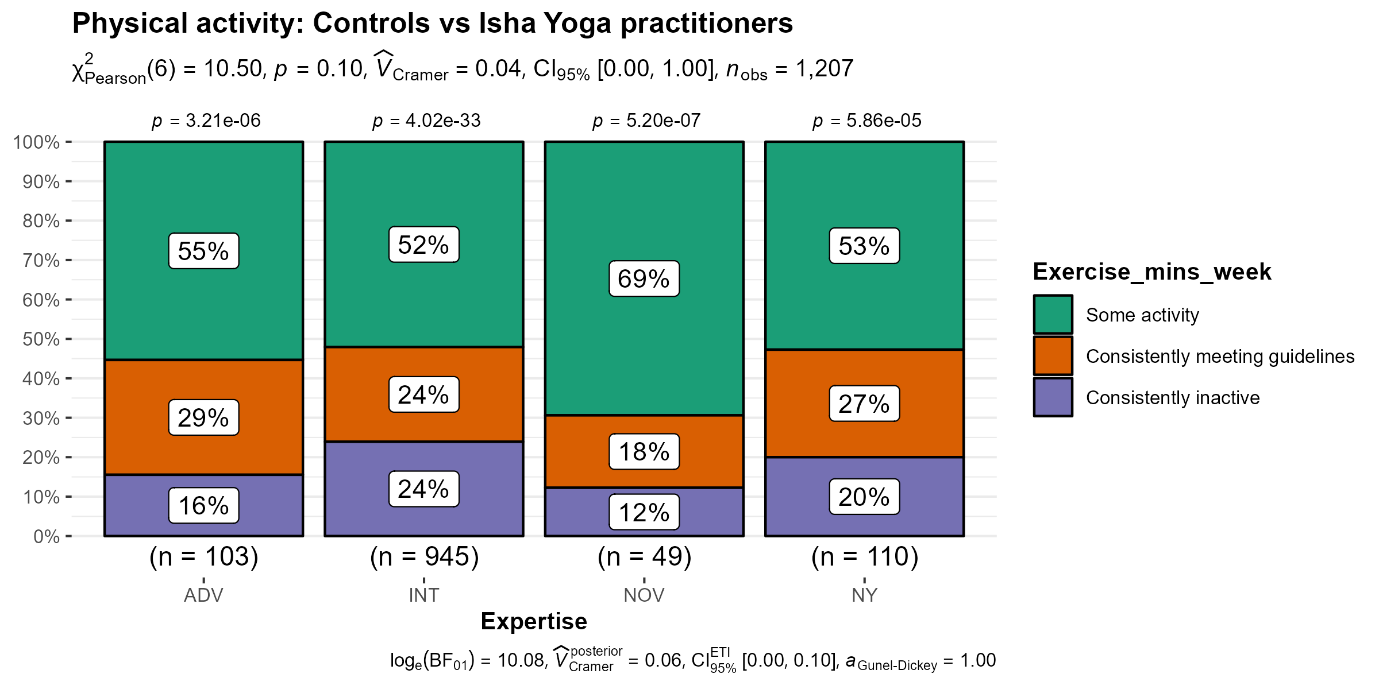


**Supplementary Figure 15:** Differences in physical activity levels between the Isha meditators and controls. ADV: Advanced Isha practitioners. INT: Intermediate Isha practitioners. NOV: Novice Isha practitioners. NY: No Yoga control group.

-

**Covid-19 Survey for Isha Yoga meditators**

**Yoga, Wellbeing and Covid-19**

The aim of this survey is to assess the impact of Isha Yoga practices on Mental Health and Wellbeing during these challenging times. Kindly fill this survey ONLY if you - 1) Are above the age of 18 years 2) Practice no other form/school of Yoga/meditation other than Isha Yoga 3) Are from Karnataka 4) Know English, Note: We are collecting your email address only to uniquely identify you. This information will be kept confidential and be available only with the investigating team.

1.

Email

*

2.

Do you provide consent to take part in this survey?

*

**INFORMED CONSENT:** You are being requested to participate in an online survey. We assure you that the information you provide will be kept confidential and be used for research purposes only. Your participation in this survey is optional and voluntary. The results of this survey may be published in a peer-reviewed journal while maintaining your anonymity. This survey is part of the PhD thesis of Mr. M. Saketh being carried out at the Centre for Consciousness Studies, Dept. of Neurophysiology, NIMHANS, Bengaluru, India, under the guidance of Prof. Bindu M Kutty, Prof. John P John, and Prof. Seema Mehrotra. If you need any further clarifications about the study, please contact Mr. M. Saketh at sakethreddy21@gmail.com

*Mark only one oval.*

Yes

No

Few instructions for filling the survey:

1) Please answer the survey with openness and honesty2) Kindly keep your mobile on a silent mode and sit in a place where you are undistracted 3) Set aside next 15 - 20 minutes solely for filling this survey 4) Don't think a lot about what to answer, whatever comes to your mind instantly, answer that5) Answer all the questions at one stretch

**Socio-demographic details**

3.

Name

*

4.

Please indicate your age range

*

*Mark only one oval.*

18 - 29 years old

30 - 44 years old

45 - 64 years old

65 years or above

5.

Gender

*

*Mark only one oval.*

Female

Male

Other

6.

Marital Status

*

*Mark only one oval.*

Married

Unmarried

Prefer not to say

7.

Residence (mention area, pincode)

*

Ex: Jayanagar, 560029

8.

Highest degree or level of education completed

*

*Mark only one oval.*

Less than high school

High school or some college

Bachelor's degree or equivalent

Postgraduate degree

Prefer not to say

9.

Monthly income of family (in Rupees)

*

*Mark only one oval.*

≥52,734

26,355-52,733

19,759-26,354

13,161-19,758

7,887-13,160

2,641-7,886

≤2,640

10.

Height (in cm)

*

You can also provide in feet and inches

11.

Weight (in Kg)

12.

Are you a healthcare worker?

*

Healthcare workers include doctors, nurses, emergency medical personnel, dental professionals andstudents, medical and nursing students, laboratory technicians, pharmacists, hospital volunteers, andadministrative staff

*Mark only one oval.*

Yes

No

**Questions related to daily sadhana**

13.

Which among these do you regularly practice? (at least 5 times per week)

*

Multiple options can be selected for this question.

*Check all that apply.*

Hatha Yoga (Upa Yoga, Angamardana, Surya Kriya, Yogasanas, etc.)

Shambhavi Mahamudra

Shakti Chalana Kriya

Shoonya

Samyama

Simha Kriya

Other:

14.

How often you do sadhana?

*

*Mark only one oval.*

Everyday

5 - 6 days per week

3 - 4 days per week

1 - 2 days per week

Less than four times per month

15.

For how long have you been doing your sadhana?

*

*Mark only one oval.*

Less than 1 month

1 - 3 months

3 - 6 months

6 months - 2 years

2 - 5 years

5 - 10 years

10 years or more

16.

On an average, how much time do you spend in your sadhana every day?

*

*Mark only one oval.*

Less than 30 minutes

30 mins - 1 hr

1 - 2 hrs

2 - 3 hrs

3 - 4 hrs

4 hrs and above

17.

What is your total life-time hours of sadhana?

*

Please calculate and give a rough estimate.

*Mark only one oval.*

Less than 100 hours

100 - 500 hours

500 - 1000 hours

1000 - 3000 hours

3000 - 10000 hours

10,000 hours or above

18.

How proficient do you think you are with your sadhana?

*

Proficiency means skill and expertise in whatever you are doing.

*Mark only one oval.*

Not at all proficient1234567

Very proficient

**Questions related to Covid-19, health, and life-style**

19.

Do you have any of these underlying medical conditions?

*

Multiple options can be selected for this question

*Check all that apply.*

History of cancer (primary and metastatic)

Chronic kidney disease

Lung disease (called as chronic obstructive pulmonary disease or COPD)

Heart disease (including myocardial infarction, congestive heart failure, peripheralvascular disease and cerebrovascular disease)

History of organ transplant

Obesity (body mass index (BMI) 30–39 kg/m2)

Class 3 obesity (BMI ≥ 40 kg/m2)

Pregnancy

Diabetes

History of blood pressure (BP)

Emergency department visit or hospitalization in the past 1 year

Diagnosed psychiatric or neurological illness

I don't have any medical condition

Other:

20.

Do you use medications for any medical condition currently?

*

*Mark only one oval.*

Yes

No

21.

Currently, how do you take care of your health and wellbeing?

*

Multiple options can be selected for this question.

*Check all that apply.*

Doing Mind and Body practices like Yoga, Meditation, Pranayama, etc

Engaging in physical activity (exercise, sports, gardening, walking, etc.)

Staying connected with friends and family members

Spending time in something I am passionate about (painting, books, cooking, music,etc.)

Other:

22.

In the last one month, on average, how many days per week did you engage in moderate to strenuous exercise (like a brisk walk)?

*

This could be anything like brisk walking, running, playing sports, cycling, dancing, etc. Please note that Hatha Yoga is NOT included in this category.

*Mark only one oval.*01234567

23.

On average, how many minutes did you engage in exercise at this level?

*

*Mark only one oval.*

0 mins/week

10 mins/week

20 mins/week

30 mins/week

40 mins/week

50 mins/week

60 mins/week

90 mins/week

120 mins/week

150 mins/week or greater

24.

What is your diet typically like?

*

*Mark only one oval.*

Vegan (no animal-based products) - At least 30% raw food

Vegan (no animal-based products) - Mostly cooked food

Vegetarian, includes dairy products (like curd, ghee) but no eggs - At least 30% raw food

Vegetarian, includes dairy products (like curd, ghee) but no eggs - Mostly cooked food

Vegetarian, includes eggs and dairy, no meat

Predominantly vegetarian, meat and eggs occasionally

Meat/veggies - meat a few times a week, eggs almost daily

Meat everyday

25.

In the last one month, how well did you sleep on an average?

*

Please rate on a scale from 1 to 7 how well you sleep on an average

*Mark only one oval.*

Poor sleep1234567

Well-rested sleep

26.

In the last one month, how many hours did you sleep every day on an average?

*

*Mark only one oval.*

Less than 4 hours per day

4 - 5 hours per day

5 - 6 hours per day

6 - 7 hours per day

7 - 8 hours per day

More than 8 hours per day

27.

Do you drink alcohol?

*

*Mark only one oval.*

Yes

No

Social drinker

28.

Do you smoke?

*

*Mark only one oval.*

Yes

No

29.

Did you use any substances in the last 6 months?

*

*Mark only one oval.*

Yes

No

30.

Have you been tested positive for Covid-19?

*

*Mark only one oval.*

Yes

No

31.

If yes to the above question, how have been the symptoms during Covid-19infection?

*

*Mark only one oval.*

Mild

Moderate

Severe

Very severe

Not applicable, I have not been tested positive

32.

(If tested positive) Have you been hospitalized because of Covid-19?

*

*Mark only one oval.*

Yes

No

Not applicable, I have not been tested positive

33.

(If tested positive) Have you been admitted to an Intensive care unit (ICU)because of Covid-19?

*

*Mark only one oval.*

Yes

No

Not applicable, I have not been tested positive

34.

(If tested positive) How has been the recovery from Covid-19?

Please rate this on a scale from 1 to 7

*Mark only one oval.*

Recovery was very slow, symptoms still exist1234567

Recovered quickly

35.

Are you vaccinated?

*

*Mark only one oval.*

Yes - first dose is completed

Yes - both doses are completed

No

**Questions related to your way of being**

These questions are related to the way "you are" generally. Please be open and honest in your responses. Rate these on a scale from 1 to 7. Example: For the first question, how peaceful are you? 1 indicates not at all peaceful, 7 indicates very peaceful, you could consider 4 to be moderately peaceful. Based on this, rate your response to the question on a scale from 1 to 7. The same logic applies to all the questions in this section.

36.

How peaceful are you?

*

Please rate this on a scale from 1 to 7

*Mark only one oval.*

Not at all peaceful1234567

Very peaceful

37.

To what extent do you prefer solitude?

*

Please rate on a scale from 1 to 7 to what extent you prefer being alone.

*Mark only one oval.*

I don't like to spend time alone; I find it stressful to handle my mental activity.1234567

I cherish spending time

38.

To what extent do you connect with people around you?

*

Please rate in the range 1 to 7 to what extent you connect to people.

*Mark only one oval.*

I find it difficult to connect to people1234567

I connect easily with people

39.

To what extent do you stay focused on what's happening in the present moment?

*

Please rate this on a scale from 1 to 7

*Mark only one oval.*

Not at all focused, my mind wanders most of the time.1234567

Totally focused and involved in whatever I am

40.

How equanimous and balanced are you?

*

Please rate this on a scale from 1 to 7

*Mark only one oval.*

Not at all equanimous1234567

Totally equanimous, no external situation affects my mind

41.

How grateful are you for your life?

*

Please rate this on a scale from 1 to 7

*Mark only one oval.*

Not at all grateful1234567

Very grateful

42.

How joyful are you?

*

Please rate this on a scale from 1 to 7

*Mark only one oval.*

Not at all joyful1234567

Very joyful

43.

How compassionate are you with everyone around you?

*

Please rate on a scale from 1 to 7 how compassionate you are with people around you.

*Mark only one oval.*

Not compassionate1234567

Very compassionate

44.

To what extent do you respect everyone regardless of their background?

*

Please rate this on a scale from 1 to 7

*Mark only one oval.*

Not at all1234567

Totally

45.

How fulfilled are you with your life?

*

Please rate on a scale from 1 to 7 how fulfilling your life is.

*Mark only one oval.*

I have no fulfillment in my life1234567

My life is very fulfilling

46.

To what extent are you handling the challenges of the Covid-19 pandemic?

*

Please rate this on a scale from 1 to 7

*Mark only one oval.*

Not able to handle this situation well1234567

Able to handle this situation very well

47.

To what extent are Yogic practices helping you deal with the challenges of theCovid-19 pandemic?

*

Please rate this on a scale from 1 to 7

*Mark only one oval.*

Not at all1234567

To a great extent

**Questions related to mental health and wellbeing**

Please indicate for each of the 5 statements which is closest to how you have beenfeeling over the past 2 weeks. Please be open and honest in your responding. Donot deliberate upon your answer. What first enters your mind, answer that.

48.

I have felt cheerful and in good spirits

*

*Mark only one oval.*

All of the time

Most of the time

More than half the time

Less than half the time

Some of the time

No time

49.

I have felt calm and relaxed

*

*Mark only one oval.*

All of the time

Most of the time

More than half the time

Less than half the time

Some of the time

No time

50.

I have felt active and vigorous

*

*Mark only one oval.*

All of the time

Most of the time

More than half the time

Less than half the time

Some of the time

No time

51.

I woke up feeling fresh and rested

*

*Mark only one oval.*

All of the time

Most of the time

More than half the time

Less than half the time

Some of the time

No time

52.

My daily life has been filled with things that interest me

*

*Mark only one oval.*

All of the time

Most of the time

More than half the time

Less than half the time

Some of the time

No time

Please think about what you have been doing and experiencing during the past four weeks. Then report how much you experienced each of the following feelings.

53.

Positive

*

*Mark only one oval.*

Very rarely or never

Rarely

Sometimes

Often

Very often or always

54.

Negative

*

*Mark only one oval.*

Very rarely or never

Rarely

Sometimes

Often

Very often or always

55.

Good

*

*Mark only one oval.*

Very rarely or never

Rarely

Sometimes

Often

Very often or always

56.

Bad

*

*Mark only one oval.*

Very rarely or never

Rarely

Sometimes

Often

Very often or always

57.

Pleasant

*

*Mark only one oval.*

Very rarely or never

Rarely

Sometimes

Often

Very often or always

58.

Unpleasant

*

*Mark only one oval.*

Very rarely or never

Rarely

Sometimes

Often

Very often or always

59.

Happy

*

*Mark only one oval.*

Very rarely or never

Rarely

Sometimes

Often

Very often or always

60.

Sad

*

*Mark only one oval.*

Very rarely or never

Rarely

Sometimes

Often

Very often or always

61.

Afraid

*

*Mark only one oval.*

Very rarely or never

Rarely

Sometimes

Often

Very often or always

62.

Joyful

*

*Mark only one oval.*

Very rarely or never

Rarely

Sometimes

Often

Very often or always

63.

Angry

*

*Mark only one oval.*

Very rarely or never

Rarely

Sometimes

Often

Very often or always

64.

Contented

*

*Mark only one oval.*

Very rarely or never

Rarely

Sometimes

Often

Very often or always

The questions here ask you about your feelings and thoughts during the last month. Please indicate how often you felt or thought a certain way.

65.

In the last month, how often have you been upset because of something that happened unexpectedly?

*

*Mark only one oval.*

Never

Almost never

Sometimes

Fairly often

Very often

66.

In the last month, how often have you felt that you were unable to control the important things in your life?

*

*Mark only one oval.*

Never

Almost never

Sometimes

Fairly often

Very often

67.

In the last month, how often have you felt nervous and “stressed”?

*

*Mark only one oval.*

Never

Almost never

Sometimes

Fairly often

Very often

68.

In the last month, how often have you felt confident about your ability to handle your personal problems?

*

*Mark only one oval.*

Never

Almost never

Sometimes

Fairly often

Very often

69.

In the last month, how often have you felt that things were going your way?

*

*Mark only one oval.*

Never

Almost never

Sometimes

Fairly often

Very often

70.

In the last month, how often have you found that you could not cope with all thethings that you had to do?

*

*Mark only one oval.*

Never

Almost never

Sometimes

Fairly often

Very often

71.

In the last month, how often have you been able to control irritations in your life?

*

*Mark only one oval.*

Never

Almost never

Sometimes

Fairly often

Very often

72.

In the last month, how often have you felt that you were on top of things?

*

*Mark only one oval.*

Never

Almost never

Sometimes

Fairly often

Very often

73.

In the last month, how often have you been angered because of things that were outside of your control?

*

*Mark only one oval.*

Never

Almost never

Sometimes

Fairly often

Very often

74.

In the last month, how often have you felt difficulties were piling up so high that you could not overcome them?

*

*Mark only one oval.*

Never

Almost never

Sometimes

Fairly often

Very often

Below are 8 statements with which you may agree or disagree. Indicate your agreement with each item by indicating that response for each statement.

75.

I lead a purposeful and meaningful life

*

*Mark only one oval.*

Strongly agree

Agree

Slightly agree

Neither agree nor disagree

Slightly disagree

Disagree

Strongly disagree

76.

My social relationships are supportive and rewarding

*

*Mark only one oval.*

Strongly agree

Agree

Slightly agree

Neither agree nor disagree

Slightly disagree

Disagree

Strongly disagree

77.

I am engaged and interested in my daily activities

*

*Mark only one oval.*

Strongly agree

Agree

Slightly agree

Neither agree nor disagree

Slightly disagree

Disagree

Strongly disagree

78.

I actively contribute to the happiness and well-being of others

*

*Mark only one oval.*

Strongly agree

Agree

Slightly agree

Neither agree nor disagree

Slightly disagree

Disagree

Strongly disagree

79.

I am competent and capable in the activities that are important to me

*

*Mark only one oval.*

Strongly agree

Agree

Slightly agree

Neither agree nor disagree

Slightly disagree

Disagree

Strongly disagree

80.

I am a good person and live a good life

*

*Mark only one oval.*

Strongly agree

Agree

Slightly agree

Neither agree nor disagree

Slightly disagree

Disagree

Strongly disagree

81.

I am optimistic about my future

*

*Mark only one oval.*

Strongly agree

Agree

Slightly agree

Neither agree nor disagree

Slightly disagree

Disagree

Strongly disagree

82.

People respect me

*

*Mark only one oval.*

Strongly agree

Agree

Slightly agree

Neither agree nor disagree

Slightly disagree

Disagree

Strongly disagree

Over the last two weeks, how often have you been bothered by the following problems?

83.

Feeling nervous, anxious or on edge

*

*Mark only one oval.*

Not at all

Several days

More than half the days

Nearly every day

84.

Not being able to stop or control worrying

*

*Mark only one oval.*

Not at all

Several days

More than half the days

Nearly every day

85.

Feeling down, depressed or hopeless

*

*Mark only one oval.*

Not at all

Several days

More than half the days

Nearly every day

86.

Little interest or pleasure in doing things

*

*Mark only one oval.*

Not at all

Several days

More than half the days

Nearly every day

Time of filling the survey

87.

When did you fill the survey?

*

*Mark only one oval.*

Before 10 am

Between 10 and 2 pm

Between 2 and 6 pm

Between 6 and 10 pm

After 10 pm

**Covid-19 Survey for people nominated by Isha meditators**

**Health, Wellbeing and Covid-19**

The aim of this survey is to assess your overall Health and Wellbeing during these challenging times. Kindly fill this survey ONLY if you - 1) Are above the age of 18 years2) Reside in Bangalore3) Know English. Note: We are collecting your email address only to uniquely identify you. This information will be kept confidential and be available only with the investigating team.

1.

Email

*

2.

Do you provide consent to take part in this survey?

*

**INFORMED CONSENT:** You are being requested to participate in an online survey. We assure you that the information you provide will be kept confidential and be used for research purposes only. Your participation in this survey is optional and voluntary. The results of this survey may be published in a peer-reviewed journal while maintaining your anonymity. This survey is part of the PhD thesis of Mr. M. Saketh being carried out at the Centre for Consciousness Studies, Dept. of Neurophysiology, NIMHANS, Bengaluru, India, under the guidance of Prof. Bindu M Kutty, Prof. John P John, and Prof. Seema Mehrotra. If you need any further clarifications about the study, please contact Mr. M. Saketh at sakethreddy21@gmail.com.

*Mark only one oval.*

Yes

No

**Few instructions for filling the survey:**

1) Please answer the survey, preferably, before 10 am 2) Kindly keep your mobile on a silent mode and sit in a place where you are undistracted 3) Set aside next 20 - 30 minutes solely for filling this survey 4) Please answer with as much openness and honesty as possible. There are no right or wrong answers for any questions. 5) Don't think a lot about what to answer, whatever comes to your mind instantly, answer that 6) Answer all the questions at one stretch

Socio-demographic details

3.

Name

*

4.

Please indicate your age range

*

*Mark only one oval.*

18 - 29 years old

30 - 44 years old

45 - 64 years old

65 years or above

5.

Gender

*

*Mark only one oval.*

Female

Male

Other

6.

Marital Status

*

*Mark only one oval.*

Married

Unmarried

Prefer not to say

7.

Residence (mention area, pincode)

*

Ex: Lakkasandra, Bangalore, 560029

8.

Highest degree or level of education completed

*

*Mark only one oval.*

Less than high school

High school or some college

Bachelor's degree or equivalent

Postgraduate degree

Prefer not to say

9.

Monthly income of family (in Rupees)

*

*Mark only one oval.*

≥52,734

26,355-52,733

19,759-26,354

13,161-19,758

7,887-13,160

2,641-7,886

≤2,640

Prefer not to say

10.

Height (in cm)

*

You can also provide in feet and inches

11.

Weight (in Kg)

*

12.

Are you a healthcare worker?

*

Healthcare workers include doctors, nurses, emergency medical personnel, dental professionals and students, medical and nursing students, laboratory technicians, pharmacists, hospital volunteers, and administrative staff

*Mark only one oval.*

Yes

No

**Questions related to Health, Life-style, and Covid-19**

13.

Do you have any of these underlying medical conditions?

*

Multiple options can be selected for this question

*Check all that apply.*

History of cancer (primary and metastatic)

Chronic kidney disease

Lung disease (called as chronic obstructive pulmonary disease or COPD)

Heart disease (including myocardial infarction, congestive heart failure, peripheral vascular disease and cerebrovascular disease)

History of organ transplant

Obesity (body mass index (BMI) 30–39 kg/m2)

Class 3 obesity (BMI ≥ 40 kg/m2)

Pregnancy

Diabetes

History of blood pressure (BP)

Emergency department visit or hospitalization in the past 1 year

Diagnosed psychiatric or neurological illness

I don't have any medical condition

Other:

14.

Do you use medications for any medical condition currently?

*

*Mark only one oval.*

Yes

No

15.

Currently, how do you take care of your health and wellbeing?

*

Multiple options can be selected for this question.

*Check all that apply.*

Doing Mind and Body practices like Yoga, Meditation, Pranayama, etc

Engaging in physical activity (exercise, sports, gardening, walking, etc.)

Staying connected with friends and family members

Spending time in something I am passionate about (painting, books, cooking, music, etc.)

I don't do any of these

Other:

16.

Do you practice any form of Yoga, Meditation, or Pranayama?

*

*Mark only one oval.*

Yes

No, never practiced in my life

Don't practice currently. Used to practice earlier, but for only less than a month

Don't practice currently. Used to practice earlier and it lasted more than a month

17.

If yes to the above question, how often do you practice?

*

*Mark only one oval.*

Everyday

5 - 6 days per week

3 - 4 days per week

1 - 2 days per week

Less than four times per month

Not applicable, I don't practice any of these

18.

If yes to the Yoga question, how much time do you spend every day in practicing these?

*

*Mark only one oval.*

Less than 15 mins

15 - 30 mins

30 mins - 1 hr

1 hr and above

Not applicable, I don't practice any of these

19.

If yes to the Yoga question, for how long have you been practicing these?

*

*Mark only one oval.*

Less than 1 month

1 - 3 months

3 - 6 months

6 months - 2 years

2 years and above

Not applicable, I don't practice any of these

20.

In the last one month, on average, how many days per week did you engage in moderate to strenuous exercise (like a brisk walk)?

*

This could be anything like brisk walking, running, playing sports, cycling, dancing, etc.

*Mark only one oval.*01234567

21.

On average, how many minutes did you engage in exercise at this level?

*

*Mark only one oval.*

0 mins/week

10 mins/week

20 mins/week

30 mins/week

40 mins/week

50 mins/week

60 mins/week

90 mins/week

120 mins/week

150 mins/week or greater

22.

What is your diet typically like?

*

*Mark only one oval.*

Vegan (no animal-based products) - At least 30% raw food

Vegan (no animal-based products) - Mostly cooked food

Vegetarian, includes dairy products (like curd, ghee) but no eggs - At least 30% raw food

Vegetarian, includes dairy products (like curd, ghee) but no eggs - Mostly cooked food

Vegetarian, includes eggs and dairy, no meat

Predominantly vegetarian, meat and eggs occasionally

Meat/veggies - meat a few times a week, eggs almost daily

Meat everyday

23.

In the last one month, how well did you sleep on an average?

*

Please rate on a scale from 1 to 7 how well you slept in the last one month.

*Mark only one oval.*

Poor sleep1234567

Well-rested sleep

24.

In the last one month, how many hours did you sleep every day on an average?

*

*Mark only one oval.*

Less than 4 hours per day

4 - 5 hours per day

5 - 6 hours per day

6 - 7 hours per day

7 - 8 hours per day

More than 8 hours per day

25.

Do you drink alcohol?

*

*Mark only one oval.*

Yes

No

Social drinker

26.

Do you smoke?

*

*Mark only one oval.*

Yes

No

27.

Did you use any substances in the last 6 months?

*

*Mark only one oval.*

Yes

No

28.

Have you been tested positive for Covid-19?

*

*Mark only one oval.*

Yes

No

29.

If yes to the above question, how have been the symptoms during Covid-19 infection?

*

*Mark only one oval.*

Mild

Moderate

Severe

Very severe

Not applicable, I have not been tested positive

30.

(If tested positive) Have you been hospitalized because of Covid-19?

*

*Mark only one oval.*

Yes

No

Not applicable, I have not been tested positive

31.

(If tested positive) Have you been admitted to an Intensive care unit (ICU) because of Covid-19?

*

*Mark only one oval.*

Yes

No

Not applicable, I have not been tested positive

32.

(If tested positive) How has been the recovery from Covid-19?

*

Please rate this on a scale from 1 to 7

*Mark only one oval.*

Recovery was very slow, symptoms still exist1234567

Recovered quickly

33.

Are you vaccinated?

*

*Mark only one oval.*

Yes - first dose is completed

Yes - both doses are completed

No

**Questions related to your way of being**

These questions are related to the way "you are" generally. Please be open and honest in your responses. Rate these on a scale from 1 to 7. Example: For the first question, how peaceful are you? 1 indicates not at all peaceful, 7 indicates very peaceful, you could consider 4 to be moderately peaceful. Based on this, rate your response to the question on a scale from 1 to 7. The same logic applies to all the questions in this section.

34.

How peaceful are you?

*

Please rate this on a scale from 1 to 7

*Mark only one oval.*

Not at all peaceful1234567

Very peaceful

35.

To what extent do you prefer solitude?

*

Please rate on a scale from 1 to 7 to what extent you prefer being alone.

*Mark only one oval.*

I don't like to spend time alone, I find it stressful to handle my mental activity.1234567

I cherish spending time

36.

To what extent do you connect with people around you?

*

Please rate in the range 1 to 7 to what extent you connect to people.

*Mark only one oval.*

I find it difficult to connect to people1234567

I connect easily with people

37.

To what extent do you stay focused on what's happening in the present moment?

*

Please rate this on a scale from 1 to 7

*Mark only one oval.*

Not at all focused, my mind wanders most of the time.1234567

Totally focused and involved in whatever I am

38.

How equanimous and balanced are you?

*

Please rate this on a scale from 1 to 7

*Mark only one oval.*

Not at all equanimous1234567

Totally equanimous, no external situation affects my mind

39.

How grateful are you for your life?

*

Please rate this on a scale from 1 to 7

*Mark only one oval.*

Not at all grateful1234567

Very grateful

40.

How joyful are you?

*

Please rate this on a scale from 1 to 7

*Mark only one oval.*

Not at all joyful1234567

Very joyful

41.

How compassionate are you with everyone around you?

*

Please rate on a scale from 1 to 7 how compassionate you are with people around you.

*Mark only one oval.*

Not compassionate1234567

Very compassionate

42.

To what extent do you respect everyone regardless of their background?

*

Please rate on a scale from 1 to 7 how you respect everyone around you.

*Mark only one oval.*

Not at all1234567

Totally

43.

How fulfilled are you with your life?

*

Please rate on a scale from 1 to 7 how fulfilling your life is.

*Mark only one oval.*

I have no fulfillment in my life1234567

My life is very fulfilling

44.

To what extent are you handling the challenges of the Covid-19 pandemic?

*

Please rate this on a scale from 1 to 7

*Mark only one oval.*

I am not able to handle this situation well1234567

I am able to handle this situation very well

45.

To what extent are your health and wellbeing practices* helping you deal with the challenges of the Covid-19 pandemic?

*

* Health and wellbeing practices include mind and body practices (like Yoga and meditation), physical activity (like walking), connecting to friends, etc.

*Mark only one oval.*

Not at all

To a great extent

**Questions related to mental health and wellbeing**

Please indicate for each of the 5 statements which is closest to how you have been feeling over the past 2 weeks. Please be open and honest in your responding. Do not deliberate upon your answer. What first enters your mind, answer that.

46.

I have felt cheerful and in good spirits

*

*Mark only one oval.*

All of the time

Most of the time

More than half the time

Less than half the time

Some of the time

No time

47.

I have felt calm and relaxed

*

*Mark only one oval.*

All of the time

Most of the time

More than half the time

Less than half the time

Some of the time

No time

48.I have felt active and vigorous

*

*Mark only one oval.*

All of the time

Most of the time

More than half the time

Less than half the time

Some of the time

No time

49.

I woke up feeling fresh and rested

*

*Mark only one oval.*

All of the time

Most of the time

More than half the time

Less than half the time

Some of the time

No time

50.

My daily life has been filled with things that interest me

*

*Mark only one oval.*

All of the time

Most of the time

More than half the time

Less than half the time

Some of the time

No time

Please think about what you have been doing and experiencing during the past four weeks. Then report how much youexperienced each of the following feelings.

51.

Positive

*

*Mark only one oval.*

Very rarely or never

Rarely

Sometimes

Often

Very often or always

52.

Negative

*

*Mark only one oval.*

Very rarely or never

Rarely

Sometimes

Often

Very often or always

53.

Good

*

*Mark only one oval.*

Very rarely or never

Rarely

Sometimes

Often

Very often or always

54.

Bad

*

*Mark only one oval.*

Very rarely or never

Rarely

Sometimes

Often

Very often or always

55.

Pleasant

*

*Mark only one oval.*

Very rarely or never

Rarely

Sometimes

Often

Very often or always

56.

Unpleasant

*

*Mark only one oval.*

Very rarely or never

Rarely

Sometimes

Often

Very often or always

57.

Happy

*

*Mark only one oval.*

Very rarely or never

Rarely

Sometimes

Often

Very often or always

58.

Sad

*

*Mark only one oval.*

Very rarely or never

Rarely

Sometimes

Often

Very often or always

59.

Afraid

*

*Mark only one oval.*

Very rarely or never

Rarely

Sometimes

Often

Very often or always

60.

Joyful

*

*Mark only one oval.*

Very rarely or never

Rarely

Sometimes

Often

Very often or always

61.

Angry

*

*Mark only one oval.*

Very rarely or never

Rarely

Sometimes

Often

Very often or always

62.

Contented

*

*Mark only one oval.*

Very rarely or never

Rarely

Sometimes

Often

Very often or always

The questions here ask you about your feelings and thoughts during the last month. Please indicate how often you felt or thought a certain way.

63.

In the last month, how often have you been upset because of something that happened unexpectedly?

*

*Mark only one oval.*

Never

Almost never

Sometimes

Fairly often

Very often

64.

In the last month, how often have you felt that you were unable to control the important things in your life?

*

*Mark only one oval.*

Never

Almost never

Sometimes

Fairly often

Very often

65.

In the last month, how often have you felt nervous and “stressed”?

*

*Mark only one oval.*

Never

Almost never

Sometimes

Fairly often

Very often

66.

In the last month, how often have you felt confident about your ability to handle your personal problems?

*

*Mark only one oval.*

Never

Almost never

Sometimes

Fairly often

Very often

67.

In the last month, how often have you felt that things were going your way?

*

*Mark only one oval.*

Never

Almost never

Sometimes

Fairly often

Very often

68.

In the last month, how often have you found that you could not cope with all the things that you had to do?

*

*Mark only one oval.*

Never

Almost never

Sometimes

Fairly often

Very often

69.

In the last month, how often have you been able to control irritations in your life?

*

*Mark only one oval.*

Never

Almost never

Sometimes

Fairly often

Very often

70.

In the last month, how often have you felt that you were on top of things?

*

*Mark only one oval.*

Never

Almost never

Sometimes

Fairly often

Very often

71.

In the last month, how often have you been angered because of things that were outside of your control?

*

*Mark only one oval.*

Never

Almost never

Sometimes

Fairly often

Very often

72.

In the last month, how often have you felt difficulties were piling up so high that you could not overcome them?

*

*Mark only one oval.*

Never

Almost never

Sometimes

Fairly often

Very often

Below are 8 statements with which you may agree or disagree. Indicate your agreement with each item by indicating that response for each statement.

73.

I lead a purposeful and meaningful life

*

*Mark only one oval.*

Strongly agree

Agree

Slightly agree

Neither agree nor disagree

Slightly disagree

Disagree

Strongly disagree

74.

My social relationships are supportive and rewarding

*

*Mark only one oval.*

Strongly agree

Agree

Slightly agree

Neither agree nor disagree

Slightly disagree

Disagree

Strongly disagree

75.

I am engaged and interested in my daily activities

*

*Mark only one oval.*

Strongly agree

Agree

Slightly agree

Neither agree nor disagree

Slightly disagree

Disagree

Strongly disagree

76.

I actively contribute to the happiness and well-being of others

*

*Mark only one oval.*

Strongly agree

Agree

Slightly agree

Neither agree nor disagree

Slightly disagree

Disagree

Strongly disagree

77.

I am competent and capable in the activities that are important to me

*

*Mark only one oval.*

Strongly agree

Agree

Slightly agree

Neither agree nor disagree

Slightly disagree

Disagree

Strongly disagree

78.

I am a good person and live a good life

*

*Mark only one oval.*

Strongly agree

Agree

Slightly agree

Neither agree nor disagree

Slightly disagree

Disagree

Strongly disagree

79.

I am optimistic about my future

*

*Mark only one oval.*

Strongly agree

Agree

Slightly agree

Neither agree nor disagree

Slightly disagree

Disagree

Strongly disagree

80.

People respect me

*

*Mark only one oval.*

Strongly agree

Agree

Slightly agree

Neither agree nor disagree

Slightly disagree

Disagree

Strongly disagree

Over the last two weeks, how often have you been bothered by the following problems?

81.

Feeling nervous, anxious or on edge

*

*Mark only one oval.*

Not at all

Several days

More than half the days

Nearly every day

82.

Not being able to stop or control worrying

*

*Mark only one oval.*

Not at all

Several days

More than half the days

Nearly every day

83.

Feeling down, depressed or hopeless

*

*Mark only one oval.*

Not at all

Several days

More than half the days

Nearly every day

84.

Little interest or pleasure in doing things

*

*Mark only one oval.*

Not at all

Several days

More than half the days

Nearly every day

Time of filling the survey

85.

When did you fill the survey?

*

*Mark only one oval.*

Before 10 am

Between 10 and 2 pm

Between 2 and 6 pm

Between 6 and 10 pm

After 10 pm
